# Pervasive and programmed nucleosome distortion on single chromatin fibres

**DOI:** 10.1038/s41586-026-10418-6

**Published:** 2026-04-29

**Authors:** Marty G. Yang, Hannah J. Richter, Simai Wang, Colin P. McNally, Camille M. Moore, Ali Emadi, Nicole E. Harris, Simaron Dhillon, Michela Maresca, Huimin Pan, Hayden Saunders, Ruiqiao Yang, Megan S. Ostrowski, Erika C. Anderson, Elzo de Wit, Jacquelyn J. Maher, Yuhong Fan, Geeta J. Narlikar, Elphège P. Nora, Holger Willenbring, Hani Goodarzi, Vijay Ramani

**Affiliations:** 1 Gladstone Institute of Data Science and Biotechnology, Gladstone Institutes, San Francisco, CA USA; 2 Department of Biochemistry and Biophysics, University of California, San Francisco, San Francisco, CA USA; 3 Tetrad Graduate Program, University of California, San Francisco, San Francisco, CA USA; 4 Arc Institute, Palo Alto, CA USA; 5 Liver Center, University of California, San Francisco, San Francisco, CA USA; 6 Division of Gastroenterology, Department of Medicine, University of California, San Francisco, San Francisco, CA USA; 7 Division of Gene Regulation, The Netherlands Cancer Institute, Amsterdam, The Netherlands; 8 Division of Transplant Surgery, Department of Surgery, University of California, San Francisco, San Francisco, CA USA; 9 Eli and Edythe Broad Center of Regeneration Medicine and Stem Cell Research, University of California, San Francisco, San Francisco, CA USA; 10 School of Biological Sciences, Georgia Institute of Technology, Atlanta, GA USA; 11 Parker H. Petit Institute for Bioengineering and Bioscience, Georgia Institute of Technology, Atlanta, GA USA; 12 Cardiovascular Research Institute, University of California, San Francisco, San Francisco, CA USA; 13 Chan Zuckerberg BioHub, San Francisco, CA USA; 14 Present Address: Department of Clinical Genetics, Erasmus University Medical Center, Rotterdam, Netherlands; 15 Present Address: Department of Chemistry, University of California, Berkeley, Berkeley, CA USA

**Keywords:** Epigenomics, Functional genomics, Gene regulation, Bioinformatics, Genomic analysis

## Abstract

Despite decades of biochemical and structural studies of the nucleosome^1^, researchers lack genome-scale methods to determine variability in nucleosome structure along individual chromatin fibres. To address this, here we present Iteratively Defined Lengths of Inaccessibility (IDLI), a computational method that maps the single-molecule co-occupancy of structurally distinct nucleosomes, subnucleosomes and other protein–DNA interactions through long-read single-molecule footprinting^2,3^. IDLI classifies methylase-inaccessible footprints on individual chromatin fibres into (i) linker-histone-associated nucleosomes; (ii) nucleosomes with focal DNA accessibility along the nucleosome wrap; (iii) unwrapped nucleosomes; and (iv) subnucleosomal species such as hexasomes, tetrasomes and other short DNA protections. Applying IDLI to chromatin from mouse embryonic stem cells, we discover that more than 85% of nucleosomes exhibit intranucleosomally accessible DNA (nucleosome ‘distortion’). We observe epigenomic-domain- and expression-level-specific patterns of distortion, including at promoters and mouse satellite repeat sequences. Transcription factor (TF) motif occurrence correlates significantly with distinct types of distortion, and degron experiments provide evidence of direct regulation by TFs. We apply IDLI to in vitro endoderm differentiation in human induced pluripotent stem cells and primary mouse hepatocytes. In both cases, we observe distortion at pioneer TF FOXA2 binding sites, demonstrating that distortion is developmentally encoded and present in vivo. Finally, genetic experiments in mice show that a nucleosome-binding domain of FOXA2 directly affects nucleosome structure in vivo, implicating these protein–nucleosome interactions as direct mediators of distortion. Our work suggests extreme but regulated nucleosome structural variability at the single-molecule level. Furthermore, our approach offers opportunities to model TF binding, nucleosome remodelling and cell-type-specific chromatin regulation across biological contexts.

## Abstract (web summary)

An analytical pipeline called Iteratively Defined Lengths of Inaccessibility (IDLI) maps the genome-wide occupancy of a range of nucleosome types and shows that most nucleosomes exhibit programmed ‘distortion’, in which the DNA exists mainly in a partially accessible state.

## Main

Regulated DNA accessibility governs cell-type-specific transcription. DNA accessibility revolves around the octameric nucleosome, which compacts approximately 147 base pairs (bp) of DNA around two dimers of histone proteins H2A and H2B flanking a tetramer of histone proteins H3 and H4. Our canonical understanding of how chromatin accessibility regulates nuclear function is simple: nucleosomes and positive transcriptional regulatory factors compete for DNA binding, particularly at active *cis*-regulatory elements^1^. Nucleosome occupancy negatively regulates transcription, and essential coactivators modulate nucleosome occupancy through chromatin remodelling.

Work over the past three decades has challenged this simple model. The synthesis of genome-scale mapping techniques and high-resolution structural biology has revealed ‘altered’ nucleosomal structures across eukaryotic epigenomes, including hexasomes (nucleosomes missing a single H2A–H2B dimer)^4–6^, overlapping dinucleosomes (hexasome–octasome complexes)^7–9^ and other noncanonical histone-containing complexes^10^. Work from various groups has shown that primary DNA sequence can guide occupancy and modulate the stability of nucleosome-wrapped DNA^11–13^. ATP-dependent chromatin remodelling by the imitation switch (ISWI) remodeller SNF2h (ref. ^14^) and chromatin compaction by the heterochromatin factor HP1 (ref. ^15^) can rely on structural distortion of nucleosomes. Finally, hexasomes and other subnucleosomal particles may be optimal substrates for chromatin remodellers (such as INO80)^16,17^. Dynamic nucleosome composition and structure are thus physiologically relevant: nuclear regulatory processes (for example, remodelling, transcription, replication and histone chaperone activity) both generate these species and render them important marks of genomic activity.

Diverse nucleosome species must also compete with essential sequence-specific TFs, which dictate cell-type-specific transcription^18^. Understanding how nucleosomes are structured with respect to TFs requires high-resolution in vivo measurement of nucleosome structures at TF-binding sites across diverse contexts. The interaction between TFs and the nucleosome is crucial to the ‘pioneer factor’ model of TF-based regulation, which describes an essential class of TFs that have intrinsically higher affinity and specificity for nucleosomal DNA than for naked DNA^19^. Structural work has identified several unique modes by which TFs can engage reconstituted nucleosomes on DNA sequences with differing mechanical properties^20–23^. Moreover, ATP-dependent chromatin remodelling enzymes cooperate with TFs to further open pioneer TF substrates^24^, and the structure of the chromatin fibre itself has been implicated in guiding the pioneer TF search process during cellular reprogramming^25^. Understanding how pioneer TF–nucleosome interactions are regulated is fundamental to understanding the chromatin basis for cell-type specification.

Technical limitations have confounded the study of canonical and noncanonical nucleosome structures. Highly resolved in situ imaging techniques (for example, electron tomography, cryo-electron tomography^26–29^ and chromatin expansion microscopy^30^) are blind to DNA sequence, and standard genomic mapping techniques rely on digestion of the chromatin fibre^5,6,31,32^. Digestion is especially problematic when mapping subnucleosomes: a hexasome-sized fragment could be derived from a true hexasome; from a nucleosome with increased DNA accessibility between a single H2A–H2B dimer and the H3–H4 tetramer; or from transient unwrapping of DNA at nucleosomal entry and exit sites (nucleosomal breathing^33,34^). Short-read single-molecule methyltransferase (MTase) footprinting methods (for example, NoME-seq^35^, MAPit^36^ and dSMF^37^) solve some of these issues but are limited by the maximum read-length (600 bp) for Illumina sequencers, and by the use of MTases specific to GpC or CpG dinucleotides. Single-molecule long-read footprinting methods that use promiscuous adenine MTases (for example, the single-molecule adenine methylated oligonucleosome sequencing assay (SAMOSA)^2,3,38^ and Fiber-seq^39^) theoretically solve these limitations, but have not yet been used to interrogate noncanonical nucleosomes.

Here we build on computational methods previously developed by our group^3^ to nondestructively resolve the subunit architecture of chromatin fibres down to individual H2A–H2B dimers and H3–H4 tetramers. Harnessing this approach, we make a key discovery: pervasive, but regulated, nucleosome distortion across both active and inactive mammalian epigenomic regions. Computational analyses and acute depletion of TFs (such as CTCF and SOX2) in mouse embryonic stem (ES) cells reveal distinct modes by which TFs distort and position nucleosomes on chromatin fibres in self-renewing cells. Extending our approach to differentiating induced pluripotent stem (iPS) cells and primary mouse hepatocytes reveals that widespread nucleosome distortion occurs over development and in vivo. Finally, by genetically perturbing the master regulatory factor FOXA2 in hepatocytes in vivo, we show that this pioneer factor has a direct role in distorting nucleosomes at FOXA2-bound regulatory regions. Taken as a whole, our work lays the foundations for the high-throughput study of causes and consequences of nucleosome structural heterogeneity.

## Subnucleosome-level single-molecule footprinting

**Fig. 1:**
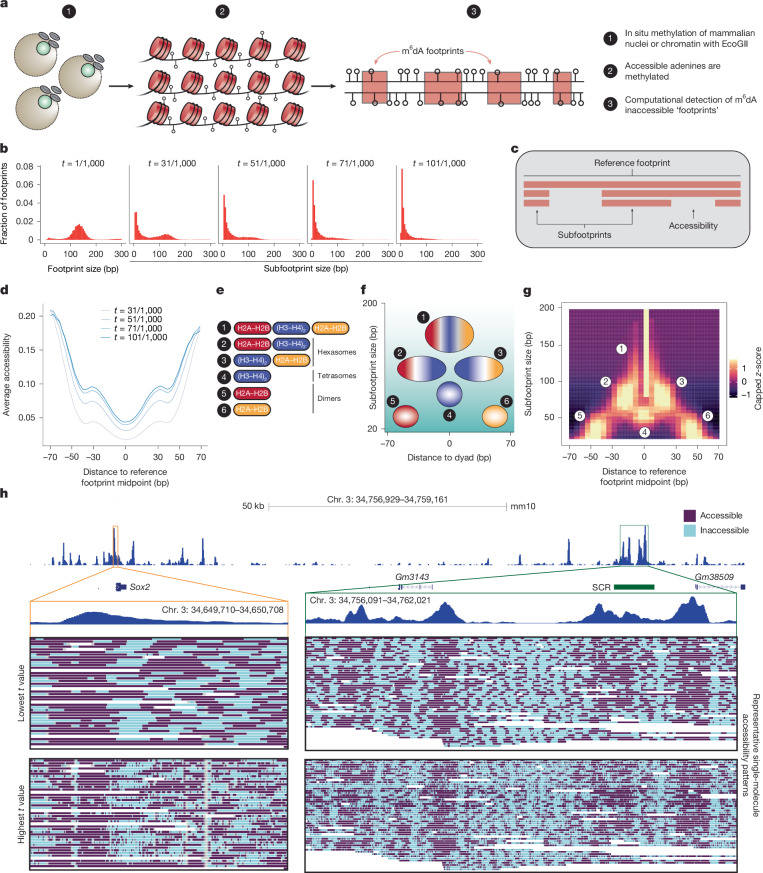
Computational framework for defining subunit architecture and organization of nucleosome core particles from in vivo long-read MTase footprinting data. **a**, Schematic of the SAMOSA assay: chromatin is methylated in situ using the EcoGII MTase, then resulting adenine-methylated (m^6^dA) DNA is sequenced on the PacBio single-molecule sequencer. **b**, Distributions of footprint and subfootprint sizes from randomly sampled footprints from E14 mouse ES cells across a range of *t* values, starting with the transition probability (*t* = 1/1,000) used in our previous work, calibrated to principally detect mononucleosome-sized footprints (*n* = 295,574 nucleosomal footprints from 34,944 sampled chromatin fibres). **c**, Schematic of a single nucleosome footprint analysed at three *t* values. Tuning the transition probability (*t*) enables analysis of subfootprint and accessibility patterns from individual footprints across different ‘resolutions’. **d**, Measured mean accessibility with respect to reference (that is, from *t* =1/1,000) footprint midpoints reveals the expected symmetric pattern about predicted dyad positions (*n* = 571,332 sampled nucleosomes). **e**, Key describing different compositions of nucleosome and subnucleosome particles, including full nucleosomes, hexasomes, tetrasomes and dimers. **f**, Predicted sizes and positions of subfootprints (plotted relative to footprint midpoints) should reflect the subunit architecture of the nucleosome, including histone octamers (1), hexasomes (lacking an H2A–H2B dimer) (2, 3), H3–H4 tetramers (4) and H2A–H2B dimers (5, 6). **g**, Horizon plot visualization (with respect to footprint midpoints) captures enrichment of subfootprint sizes and positions, including octameric nucleosomes that exhibit ‘unspooling’ of DNA from entry and exit sites (feature 1; vertical line centred at footprint midpoint) (*n* = 571,332 sampled nucleosomes). **h**, Single-molecule accessibility patterns (EcoGII-accessible and -inaccessible DNA in purple and in teal, respectively) at the *Sox2* promoter (left; orange) and the *Sox2* control region (SCR) (right; green). Data from representative single EcoGII-footprinted fibres are shown for the lowest (*t* = 1 / 1,000; top) and highest (*t* = 101 / 1,000; bottom) transition probabilities used in IDLI. Bulk-average chromatin accessibility data from ATAC-seq in mouse ES cells are shown across the entire locus (top) and at specific regulatory loci (bottom).

We previously introduced SAMOSA (schematic in Fig. 1a), which uses the nonspecific adenine MTase EcoGII and PacBio sequencing to footprint chromatin fibres genome-wide^34^. We infer protein–DNA contacts from EcoGII methylation using a neural network–two-state hidden Markov model (NN-HMM) that explicitly accounts for both sequence biases of the EcoGII enzyme and sequence biases that influence the kinetics of PacBio sequencing, to infer mononucleosome ‘footprint’ positions from the sequencing polymerase kinetics^3^.

**Extended Data Fig. 1:**
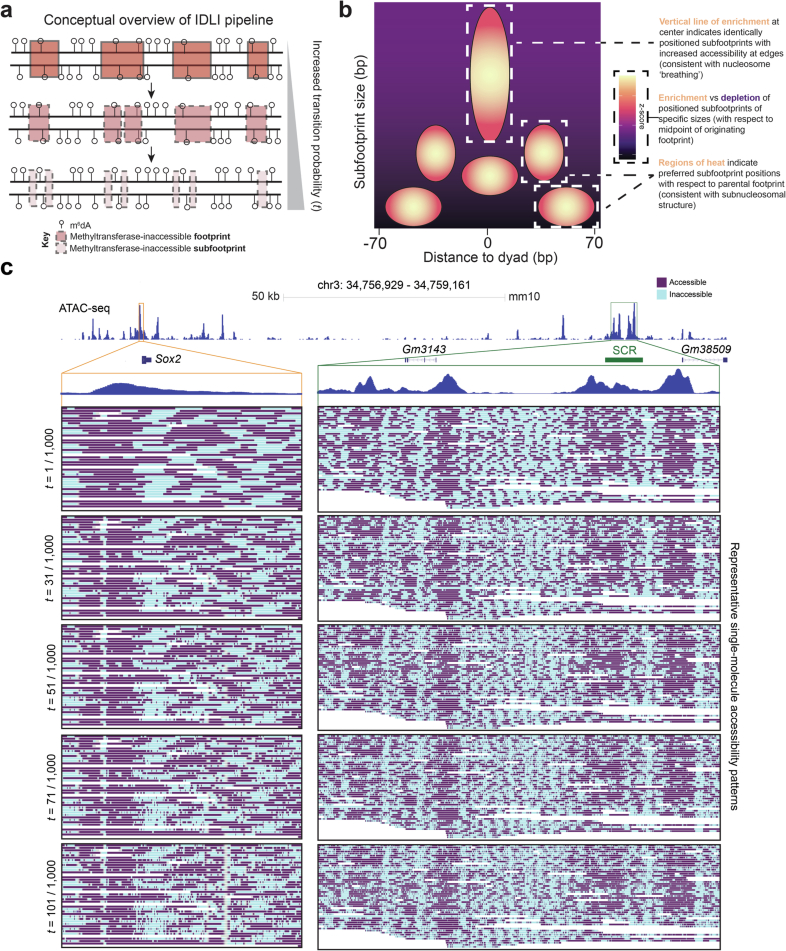
Conceptual overview of the IDLI pipeline and example multi-scale genome browser tracks. **a**, As shown in this schematic, individual footprints are subdivided into shorter ‘subfootprints’ at higher transition probabilities (‘*t*’; the likelihood of switching between the ‘inaccessible’ and ‘accessible’ model states). This enables ‘multi-scale’ analysis of nucleosomes beyond simply calling a nucleosome footprint and its respective size. **b**, Visual schematic for the ‘horizon plot’ with salient features highlighted. The horizon plot borrows conceptual elements from the ‘V-plot’ first described by Henikoff and colleagues^27^. Regions of heat indicate specific MTase-inaccessible stretches of nucleosome size, whereas footprint-internal methylation yields an enrichment of smaller, positioned ‘subfootprints’. **c**, Single-molecule accessibility patterns (EcoGII-accessible and -inaccessible DNA in purple and teal, respectively) at *Sox2* promoter (left; depicted in orange) and *Sox2* control region (SCR) (right; depicted in green). Data from representative identical single EcoGII-footprinted fibres are shown for the full range of transition probabilities used in IDLI (*t* = 1, 31, 51, 71 and 101/1,000). Bulk-average chromatin accessibility data (from ATAC-seq) in mouse ES cells are also shown across the entire locus (top) and at specific regulatory loci (bottom).

We hypothesized that our model might detect the subunit structure of mononucleosomes by varying the user-defined transition probability, hereafter, *t* (Extended Data Fig. 1a; see Methods and Supplementary Note for conceptual motivation). To test this hypothesis, we collated a large amount of SAMOSA data from the E14 mouse ES cell line (88.87 Gb total; library metadata summarized in Supplementary Table 1) and called footprints varying the *t* parameter. Plotting the distributions of called footprint sizes across a wide range of *t* values confirmed that increasing *t* results in successively smaller footprint calls (hereafter referred to as ‘subfootprints’; Fig. 1b).

We reasoned that if subfootprints were biologically ‘patterned,’ then when aligned (Fig. 1c) and plotted as footprint size versus midpoint position ‘horizon plots’ (analogous to V-plots for micrococcal nuclease sequencing (MNase-seq) data^31^), subfootprints should reflect the composition of the nucleosome. Visualization of computed accessibility at multiple *t* values (Fig. 1d) suggested that these patterns exist in the data. Horizon plot visualization of subfootprints (Fig. 1e–g) revealed a clear signal enrichment of footprints (Fig. 1g), which qualitatively corresponded to the subunit architecture of the nucleosome (Fig. 1e,f). These patterns were consistent with ‘breathing’ nucleosomes^40^ (vertical line; feature 1), hexasomes (features 2 and 3), tetramers (feature 4) and dimers (features 5 and 6; additional interpretation aid shown in Extended Data Fig. 1b). Footprint and subfootprint positions on individual fibres were visualized along bulk ATAC-seq (assay for transposase-accessible chromatin using sequencing) data^41^ at the *Sox2* locus, as an example (Fig. 1h and Extended Data Fig. 1c). Together, these results demonstrate our ability to footprint the subunit architecture of the nucleosome on individual chromatin fibres in mammalian cells. We refer to this computational strategy, which depends on iteratively scoring footprinted molecules, as Iteratively Defined Lengths of Inaccessibility, or IDLI.

## Genome-wide nucleosome distortion

**Fig. 2:**
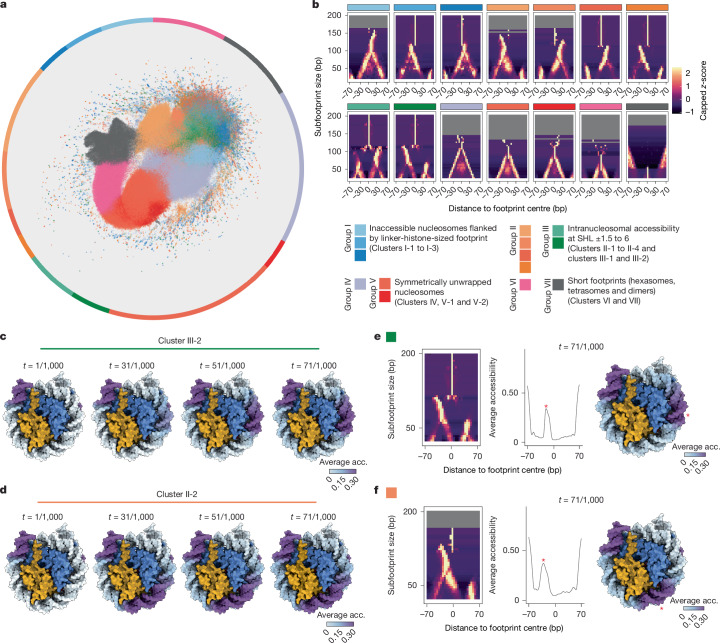
Defining structurally distinct nucleosomes using IDLI. **a**, UMAP visualization of *n* = 795,765 footprints within ±500 bp of midpoints of histone post-translational-modification-defined epigenomic domains (in mouse ES cells) or ‘unmappable’ repeat elements. Individual footprints within UMAP are coloured on the basis of Leiden-clustering assignments of their intranucleosomal accessibility (distortion patterns). Proportions of each cluster are depicted as a pie chart surrounding the UMAP plot. A key for the individual nucleosome types and respective ‘groups’ (as defined by hierarchical clustering) is shown on the right. **b**, ‘Horizon plots’ for 14 distinct structural classes of footprints. **c**,**d**, Structural visualization of how computed accessibility (acc.) changes on the nucleosome structure at different *t* values for cluster III-2 (**c**) and cluster II-2 (**d**). **e**, Horizon plot (left), mean accessibility (middle) and structural visualization (right) of cluster III-2 footprints, which are nucleosome core particles accessible at SHL ±2 (denoted by the red asterisk). **f**, As in **e**, but for cluster II-2 footprints, which are nucleosome core particles accessible at SHL ±3.5.

**Extended Data Fig. 2:**
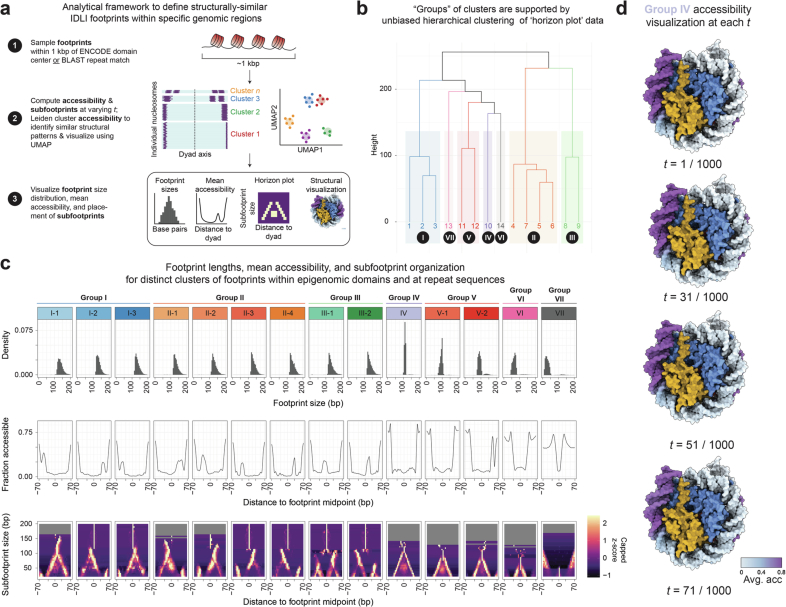
The IDLI pipeline defines nucleosome types through unbiased clustering of nucleosome accessibility patterns. **a**, Framework for clustering and visualization of footprints with shared structural features (patterns of intranucleosomal accessibility). For computational feasibility, we sample footprints specific genome regions or from repetitive elements uniquely mappable by PacBio long-read sequencing. Single-footprint accessibility patterns across *t* = 31, 51 and 71/1,000 are subject to Leiden clustering, and resulting data are visualized via UMAP. We examine Leiden clusters on the basis (1) footprint size distributions, (2) mean accessibility profiles with respect to footprint midpoints, (3) ‘horizon plots’ for detecting subfootprint placement, and (4) a structural visualization of mean accessibility overlaid on the PDB structure for an interphase nucleosome (7KBD)^44^. **b**, Clustering results in 14 Leiden-defined clusters that fall into 7 distinct groups that are grouped together on the basis of hierarchical clustering of horizon plots themselves. **c**, Analysis of individual Leiden-defined clusters, which we term ‘nucleosome types’. Top: Histograms of footprint sizes on a per-cluster basis. Middle: Mean accessibility traces centred on footprint midpoints on a per-cluster basis. Bottom: ‘Horizon plots’ for subfootprint sizes versus distance from footprint midpoints to assess subfootprint enrichment at particular locations on a per-cluster basis. z-scores are capped such that the maximum shown in the heat map is the 95th percentile of values of the heat map. **d**, Example of visualizing one nucleosome type (IV) at multiple different *t*, with computed accessibility around the nucleosome core particle shown in purple.

**Extended Data Fig. 3:**
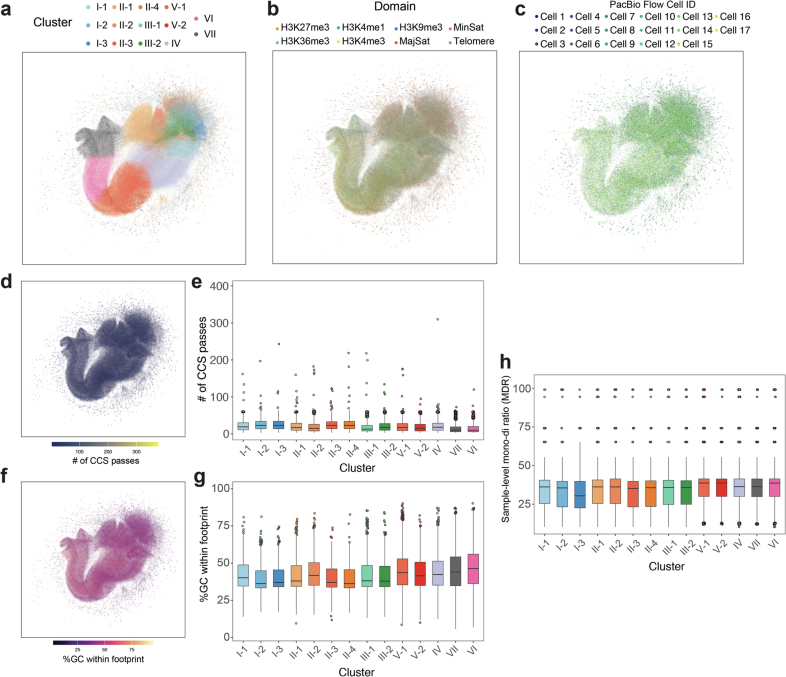
Per-nucleosome statistics for IDLI-defined nucleosome types. **a**, UMAP of clustered nucleosomes, coloured by nucleosome-type definition (naming convention: [group roman numeral] – [unique numeral per group]). **b**, Same UMAP as in **a** but colouring nucleosomes by the epigenomic domain/repeat class they fall within. **c**, Same UMAP as in **a** but colouring nucleosomes by flow cell on which the library was run. **d**, Same UMAP as in **a** but colouring nucleosomes by the number of CCS passes of the read on which the nucleosome footprint was called. **e**, Alternative visualization of data shown in **d**; box plots of number of CCS passes for each nucleosome type. **f**, Same UMAP as in **a** but colouring nucleosomes by GC content of each footprint. **g**, Alternative visualization of data shown in **f**; box plots of GC content within footprint for each nucleosome type. **h**, Box plot visualization of the mono-di ratio (computed per sample as a measure of methylation extent for a sample) for nucleosomes falling within each nucleosome type.

We used IDLI to evaluate the structural heterogeneity of nucleosomes genome-wide. We devised a classification approach (Extended Data Fig. 2a) using Leiden community detection^42^ and uniform manifold approximation and projection (UMAP) visualization^43^ algorithms to classify and visualize distinct IDLI footprints. We applied this approach to 795,765 sampled nucleosomes from 145,065 sampled E14 mouse ES cell chromatin fibres. We binned clusters into ‘groups’ through hierarchical clustering (Extended Data Fig. 2b) and examined the underlying properties of each cluster of nucleosomes. We specifically examined three properties (Extended Data Fig. 2c): (i) per-cluster footprint sizes; (ii) average accessibility of footprints at the highest *t* value used for clustering (*t* = 0.071); and (iii) per-cluster horizon plots. Our joint analysis and visualization pipeline (UMAP coloured by cluster shown in Fig. 2a) resulted in 14 clusters, which we hereafter refer to as ‘nucleosome types’ (Fig. 2b). These nucleosome types could be further clustered into seven self-similar groups (I–VII). Colouring of individual nucleosome footprints in UMAP by technical co-variates (GC content, circular consensus sequencing (CCS) passes, batch) revealed only subtle associations within the *k-*nearest neighbours graph (Extended Data Fig. 3). We interpret nucleosome types as distinct in cellulo structural nucleosomal states across the genome.

We next plotted the accessibility of different nucleosome types at various *t* values along a solved structure^44^. In some cases (for example, for nucleosome type IV; Extended Data Fig. 2d), visualization across various *t* values showed only subtle changes. In other cases, however, we observed unique, focal patterns of accessibility emerge within the nucleosome core particle at high *t* (clusters III-2 and II-2 shown in Fig. 2c,d, respectively). Closer examination of horizon plots, accessibility and structure for these specific clusters revealed focal accessibility specifically at superhelical location (SHL) ±2 (Fig. 2e) and at SHL ±3.5 (Fig. 2f). These experiments together suggest that the majority (86.6% of footprints falling into groups II–VII) of nucleosomes in E14 mouse ES cells show some degree of intranucleosomal DNA accessibility, implying pervasive nucleosome distortion genome-wide.

## Validating nucleosome types

**Fig. 3:**
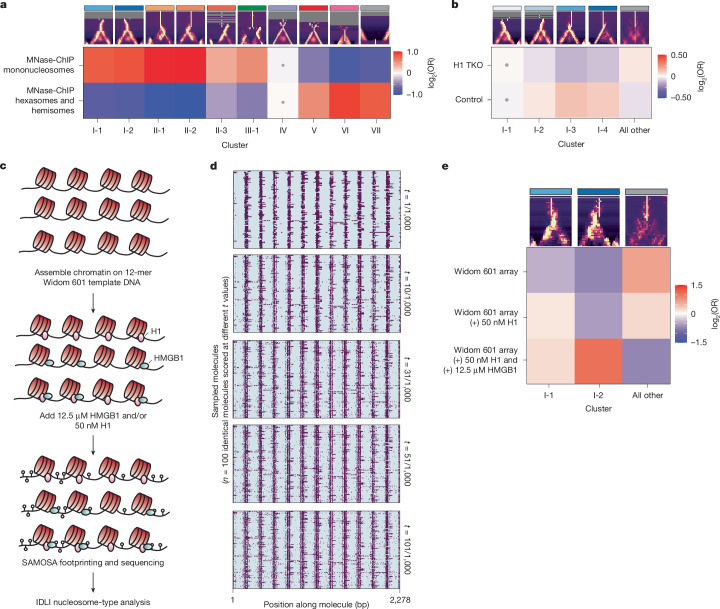
Validating nucleosome types through integrated genomics, genetics and biochemistry. **a**, Heat map depiction of enriched or depleted nucleosome types from E14 mouse ES cells, falling within regions of the mouse ES cell epigenome with greater abundance of full nucleosomes or subnucleosomes (for example, hexasomes and hemisomes), defined through biochemical fractionation or MNase-ChIP (ref. ^47^). Grey dots indicate Fisher’s exact test results that are not significant (Storey *q* > 0.1). **b**, Heat map depiction of enriched or depleted nucleosome types for nucleosomes sampled from SAMOSA experiments performed in H1 TKO mouse ES cells compared with matched isogenic controls. Grey dots indicate test results that are not significant (*q* > 0.1). **c**, To complement our genomic approach, we performed the IDLI pipeline on single-molecule footprinting data from purely reconstituted chromatin fibres bound by linker histone H1 and/or the DNA-binding protein HMGB1 (ref. ^46^). **d**, Sampled molecules from the SAMOSA to test chromatin accessibility on assembled templates (SAMOSAChAAT) footprinting experiment with 50 nM H1 and 12.5 μM HMGB1, at five example *t* values. **e**, Heat map depiction of enriched or depleted nucleosome types from the in vitro footprinting experiment stratified by sample type.

**Extended Data Fig. 4:**
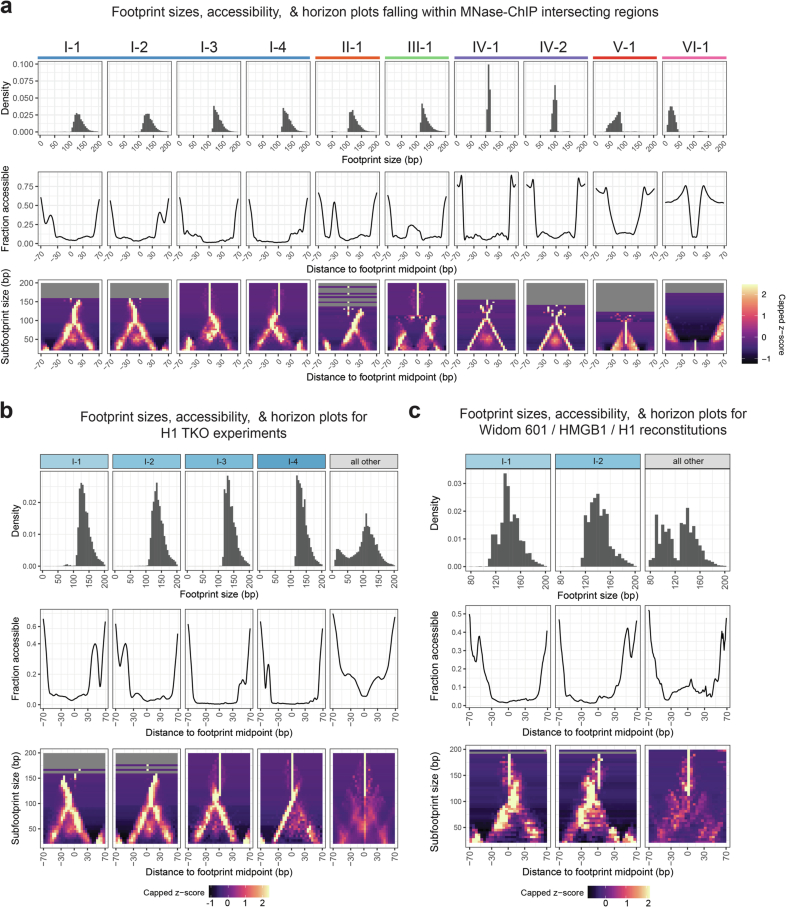
Nucleosome-type metadata for MNase-ChIP, H1 TKO and in vitro reconstitutions. **a**, Analysis of individual nucleosome types for nucleosome footprints falling within MNase-ChIP defined genomic regions enriched for subnucleosomes versus full nucleosomes. Top: Histograms of footprint sizes on a per-cluster basis. Middle: Mean accessibility traces centred on footprint midpoints on a per-cluster basis. Bottom: horizon plots for subfootprint sizes versus distance from footprint midpoints to assess subfootprint enrichment at particular locations on a per-cluster basis. z-scores are capped such that the maximum shown in the heat map is the 95th percentile of values of the heat map. **b**, As in **a** but for H1 TKO and matched control mouse ES cell data. **c**, As in **a**,**b** but for SAMOSA-ChAAT data generated from Widom 601 reconstitutions in the presence of linker histone H1 and/or recombinant HMGB1 protein.

Nucleosome–cofactor interactions^45,46^, dynamic (dis)assembly of H2A–H2B dimers and the H3–H4 tetramer and assembly of linker histone H1-containing chromatosomes could all contribute to the heterogeneity we observe. We hypothesized that nucleosome types map directly to known nucleosome species, and tested this hypothesis by applying IDLI to genomic, genetic and biochemical data. Genomically, we integrated our nucleosome call set with orthogonal MNase chromatin immunoprecipitation followed by sequencing (ChIP–seq) data also from mouse ES cells^47^, in which subnucleosomes were mapped and validated at near-nucleotide resolution. We defined nucleosome types using IDLI within 1-kb genomic windows that were enriched or depleted for these subnucleosomes (yielding a subset of the same seven broad groups; Extended Data Fig. 4a). We then computed the relative enrichment of nucleosome types falling within these windows, visualized as a heat map of effect sizes (Fig. 3a). Highly accessible nucleosome types were significantly enriched (for example, group VI odds ratio (OR) = 1.875 and Storey *q* < 1.79 × 10^−^^308^) in MNase-ChIP hexasome and hemisome fractions, suggesting that these group V–VII nucleosomes are bona fide subnucleosomal species. By contrast, the least accessible nucleosome types in groups I–III were significantly enriched in MNase-ChIP mononucleosome fractions.

We next used genetic perturbation to validate the identity of the least accessible nucleosome types. We performed SAMOSA on a mouse ES cell model in which three of six linker histone genes were genetically ablated (H1 triple knockout; TKO), resulting in a reduction of around 50% in linker histone on chromatin^48^. IDLI analysis of data from H1 TKO and matched isogenic control lines resulted in the same broad definition of nucleosome types (Extended Data Fig. 4b). Enrichment analysis of these nucleosome types revealed a significant depletion of specific group I nucleosomes, compared with other nucleosome types (Fig. 3b; for example, cluster I-3 OR = 0.913; *q* = 4.36 × 10^−04^). From this, we conclude that group I nucleosomes reflect chromatosomes that contain linker histone H1.

Finally, we validated nucleosome types using published footprinting data from biochemical reconstitutions^46^. We previously performed in vitro footprinting experiments^3^ on chromatin arrays assembled on repeats of the Widom 601 nucleosome positioning sequence, with recombinant linker histone H1 (50 nM) and the highly abundant, chromatin-interacting and HMG-box containing protein HMGB1 (12.5 µM)^46^. We applied IDLI to these data (Fig. 3c), revealing subfootprints even on nucleosomes assembled on the synthetic Widom 601 sequence (Fig. 3d). IDLI classification of nucleosome types from these in vitro experiments revealed similar patterns to those seen in cells (Extended Data Fig. 4c). For example, we observed group I nucleosomes enriched in reconstitutions that contained 50 nM linker histone H1 (Fig. 3e; for example, I-1 OR = 1.109; *q* = 1.98 × 10^−2^), and even more enriched in those that contained 50 nM H1 and 12.5 µM HMGB1 (I-2 OR = 2.075; *q* = 9.50 × 10^−65^). These results suggest that group I nucleosomes seen in vivo represent nucleosomes that are bound by both H1 and HMGB1, or another HMG-box protein. The direct correspondence of nucleosome types observed both in vitro and in vivo confirms that IDLI measures true changes in nucleosome structure, which are likely to be caused by specific protein–nucleosome interactions (for example, HMG-family proteins interacting with chromatosomes^49^).

## DNA-binding factors distort nucleosomes

**Fig. 4:**
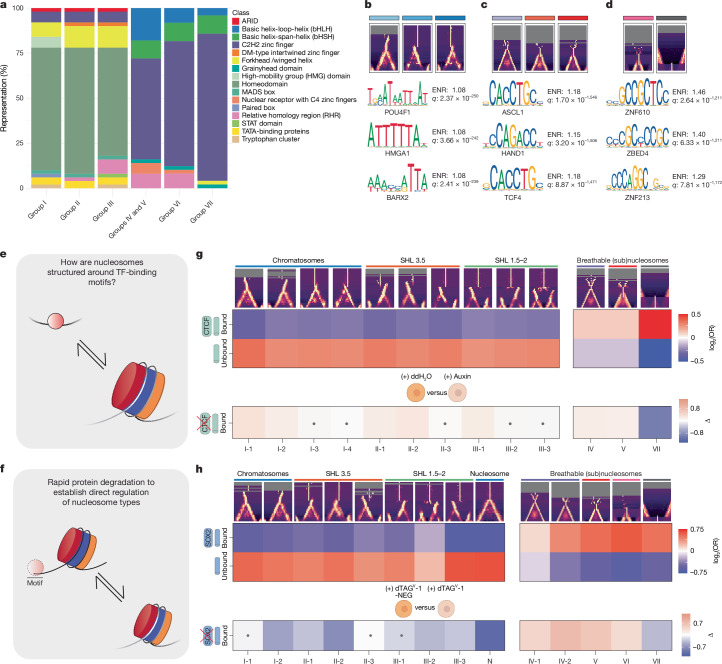
Transcription factors directly regulate nucleosome distortion. **a**, Representation of TF motif families whose motifs fall within distinct nucleosome types. **b**–**d**, Example motif matches and associated motifs, enrichment scores (ENR) and *q-*values for group I (**b**), group IV and V (**c**) and group VI and VII (**d**) nucleosome types called from E14 mouse ES cells. **e**, We sought to understand how nucleosome types are arranged around specific TF motifs, focusing on CTCF and SOX2. **f**, We further aimed to establish direct regulation using rapid conditional degradation (with either the auxin or the dTAG degron system). **g**, Heat map of log_2_-transformed ORs for assessing the enrichment (red) or depletion (blue) of structurally distinct nucleosome types at bound versus randomly sampled CTCF motifs in E14 mouse ES cells. ORs designated with a grey dot indicate those that are not statistically significant in a Fisher’s exact test (Storey *q* > 0.1). The final row of the heat map plots effect sizes (Δ) for nucleosome types at bound CTCF motifs in auxin versus ddH_2_O (control) CTCF-auxin inducible degron (AID) mouse ES cells. Effect sizes designated with a grey dot indicate those that are not statistically significant in a Fisher’s exact test (Storey *q *> 0.1). **h**, Heat map of log_2_-transformed ORs for assessing the enrichment (red) or depletion (blue) of structurally distinct nucleosome types at bound versus randomly sampled SOX2-OCT4 motifs in E14 mouse ES cells. ORs designated with a grey dot indicate those that are not statistically significant in a Fisher’s exact test (Storey *q* > 0.1). The final row of the heat map plots effect sizes (Δ) for nucleosome types at bound SOX2-OCT4 composite motifs in dTAG^V^-1 versus dTAG^V^-1-NEG-treated (control) SOX2-FKBP mouse ES cells. Effect sizes designated with a grey dot indicate those that are not statistically significant in a Fisher’s exact test (Storey *q* > 0.1). The illustrations of cell types in **g**,**h** were created in BioRender; Ramani, V. https://BioRender.com/ckmwvd1 (2026).

**Extended Data Fig. 5:**
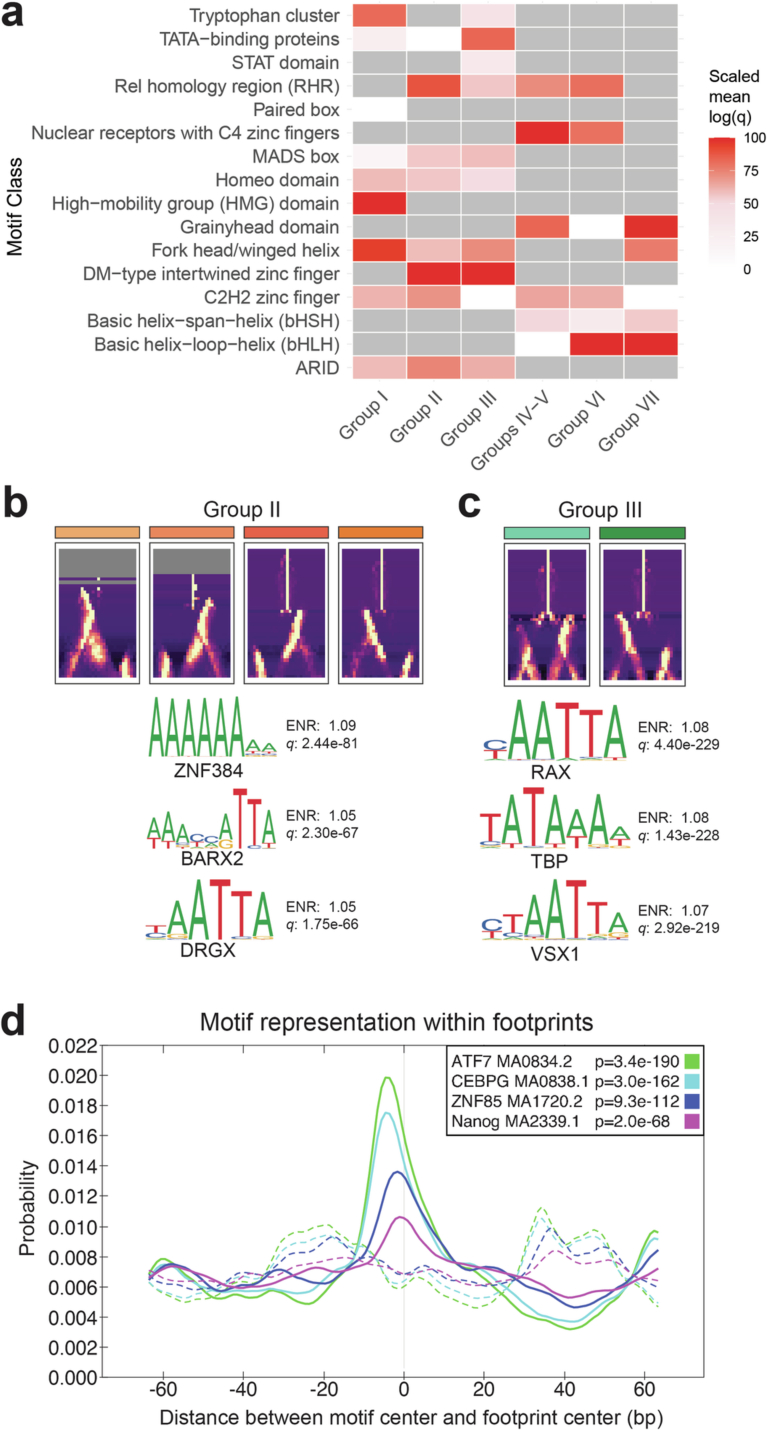
Motif enrichment and positioning across nucleosome types. **a**, Heat map representation of significance (scaled mean log(*q*)) for each motif family enrichment across each group of nucleosome types. **b**,**c**, Example motif matches and associated motifs, enrichment scores, and *q-*values for group II (**b**) and group III (**c**) nucleosome types called from E14 mouse ES cells. **d**, Distribution of motif positions for ATF7, CEBPG, ZNF85 and Nanog motifs with respect to footprint centres.

TFs must recognize their cognate DNA sequence motifs in chromatinized environments^18^. We speculated that nucleosome types are motif encoded, as previously demonstrated for pioneer TFs during endoderm specification^50^. To assess this, we performed motif analyses on E14 nucleosome types using simple enrichment analysis (SEA)^51^, and concurrently examined the representation of JASPAR-annotated TF classes^52^ among called motifs. Notably, we observed specific TF- and chromatin-associated protein motif classes enriched across different groups (Fig. 4a). Example enriched motifs, their associated enrichment scores and *q-*values are provided in Fig. 4b–d and Extended Data Fig. 5a. Group I nucleosome types were enriched for homeodomain binding motifs (olive box in Fig. 4a), with select enriched motifs including those of homeobox factors POU4F1 and BARX2, and the AT-hook motif of the chromatin-associated HMG-family protein HMGA1 (Fig. 4b). Group II and III nucleosome types were uniquely enriched for forkhead family motifs (yellow box in Fig. 4a and Extended Data Fig. 5b,c), indicating a possible mode by which pioneer factors engage nucleosomes. Group IV and V nucleosome types were enriched for E-box binding motifs (blue box in Fig. 4a and Fig. 4c), suggesting yet another mode of TF–nucleosome interaction involving symmetric nucleosome breathing. Finally, the shortest footprints classified by the IDLI pipeline (groups VI and VII; Fig. 4d) were enriched for motifs recognized by C2H2 zinc finger (ZF) class TFs (purple box in Fig. 4a); we speculate that these represent minimally protected footprints of ZF-DNA interactions on chromatin. TF motifs also showed a distribution of positions with respect to the centre of nucleosome footprints, suggesting that there is a range of docking sites along distorted nucleosomes for different TFs (see Extended Data Fig. 5d for representative TFs).

**Extended Data Fig. 6:**
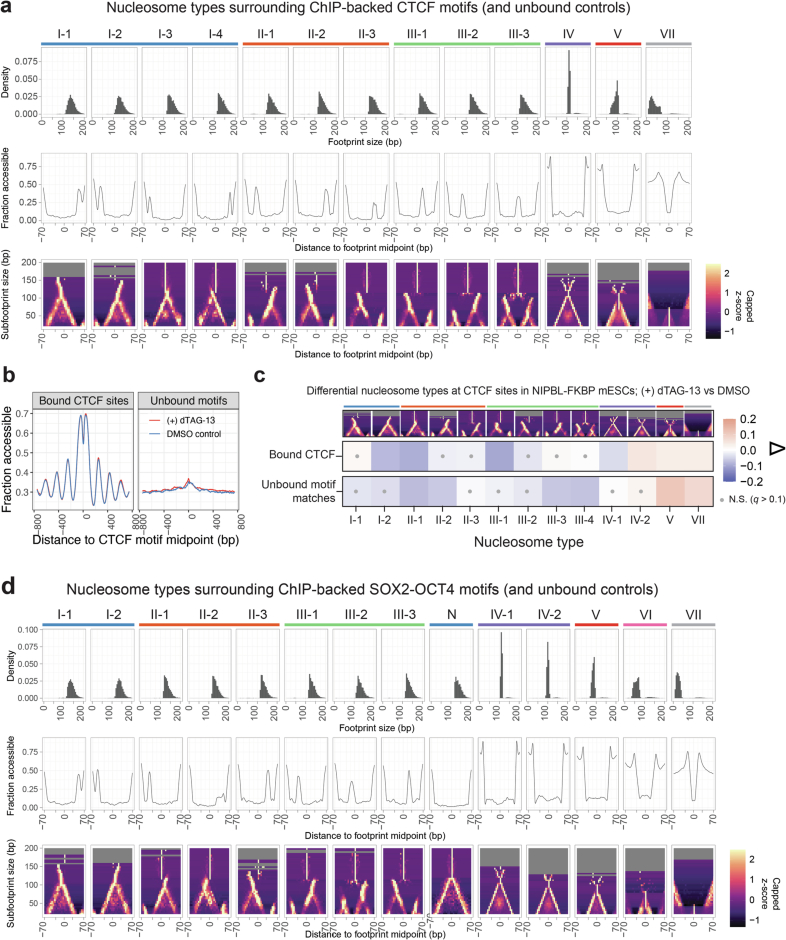
Nucleosome-type metadata for CTCF and SOX2-OCT4-focused experiments in mouse E14 ES cells, CTCF-AID degron ES cells, NIPBL-FKBP degron ES cells and SOX2-FKBP degron ES cells. **a**, Analysis of individual nucleosome types for nucleosome footprints falling in the vicinity of CTCF motifs with ChIP–seq backed evidence of binding (bound), and unbound motif matches (unbound). Top: Histograms of footprint sizes on a per-cluster basis. Middle: Mean accessibility traces centred on footprint midpoints on a per-cluster basis. Bottom: horizon plots for subfootprint sizes versus distance from footprint midpoints to assess subfootprint enrichment at particular locations on a per-cluster basis. *z*-scores are capped such that the maximum shown in the heat map is the 95th percentile of values of the heat map. **b**, Average accessibility at bound/unbound CTCF motifs in NIPBL-FKBP mouse ES cells in the presence (red) and absence (blue) of dTAG-13. **c**, Differential nucleosome-type representation at bound/unbound CTCF motifs in NIPBL-FKBP mouse ES cells (dTAG-13 versus DMSO control. Grey dots represent *q* > 0.1). Degron experiments were pooled over two time points (6 h and 24 h) to capture the full range of phenotypes downstream of NIPBL depletion. **d**, As in **a** but for SOX2-OCT4 motifs in mouse ES cells, including SOX2-FKBP mouse ES cell data.

How TFs engage their motifs in the context of nucleosomes remains contentious^18,19,53^. We reasoned that the position and identity of nucleosome types in reference to TFs could also be analysed by assessing nucleosome types at specific bound TF motifs compared with unbound control motifs (Fig. 4e), with causal links being drawn from experiments with small-molecule sensitive degrons^50^ (Fig. 4f). We first applied this approach to the CCCTC-binding factor (CTCF), an essential TF with diverse roles in the nucleus^54^. Using data from our previously published CTCF degron experiments^52^, we clustered footprints falling near bound and unbound CTCF motifs, resulting in 13 nucleosome types in 7 groups, as above (Extended Data Fig. 6a). We computed the extent to which these nucleosome types differed at ChIP-backed CTCF motifs versus random motif matches (Fig. 4g, top two rows). We observed a significant enrichment of subnucleosome and short footprints at bound CTCF motifs specifically (for example, VII OR = 1.309; *q* < 1.79 × 10^−308^), accompanied by significant depletion for less-accessible nucleosome types. Depletion of CTCF protein (Fig. 4g, bottom row) resulted in a significant loss of short footprints (degron VII OR = 0.681; *q* = 6.93 × 10^−103^). These analyses suggest that CTCF directly regulates the composition of structurally distinct nucleosomes to favour partially unwrapped states. We note that these results seem independent of CTCF’s role as a pausing factor for the cohesin holoenzyme. IDLI analyses in mouse ES cells in which the cohesin stimulatory factor NIPBL was rapidly depleted through dTAG degradation^55,56^ revealed minimal differences in single-molecule accessibility at bound versus unbound sites (Extended Data Fig. 6b). Although depletion of NIPBL did alter nucleosome-type distributions significantly (suggesting that global loop extrusion has a role in regulating nucleosome structure), the observed effects were not specific to CTCF sites (Extended Data Fig. 6c).

We next focused on the core pluripotency TF SOX2. Strong CTCF motifs are sufficient to displace well-positioned nucleosomes in cells^57^, but how SOX2 motifs contend with nucleosomes remains unclear. Genomic, structural and other biochemical assays have offered conflicting results on how SOX2 engages chromatin, particularly with regard to SOX2 interactions within the nucleosome^20,57^, versus at the nucleosome entry and exit site^21,58^. To provide molecular insight into these possible binding modes for SOX2, we repeated the analyses above at SOX2-OCT4 composite motifs^59^, including footprinting experiments in a SOX2-FKBP mouse ES cell line^60^. IDLI revealed 14 nucleosome types (Extended Data Fig. 6d). These types fell into similar groups as those described previously, with the addition of a second inaccessible nucleosome type that we termed ‘N.’ Differential analysis (Fig. 4h, top two rows) revealed a significant enrichment for breathing nucleosomes, subnucleosomes and short footprints at bound versus unbound motifs (for example, VI OR = 1.420; *q* = 1.42 × 10^−247^), as well as depletion of inaccessible nucleosome species. We speculate that these patterns reflect SOX2 directly restructuring nucleosomes at these elements, either on its own or through interaction with other chromatin regulators. As with CTCF depletion, acute degradation of SOX2 protein (Fig. 4h, bottom row) resulted in significant depletion of short footprints (degron VII OR = 0.871, *q* = 1.79 × 10^−3^), consistent with a subset of short protections representing TF-protected footprints. Paradoxically, acute depletion of SOX2 was also accompanied by a significant decrease in the proportion of inaccessible nucleosome species (for example, degron N OR = 0.713, *q* = 1.95 × 10^−5^). These results suggest that SOX2 protein stabilizes nucleosomal species in its vicinity.

## Epigenomic correlates of nucleosome types

**Fig. 5:**
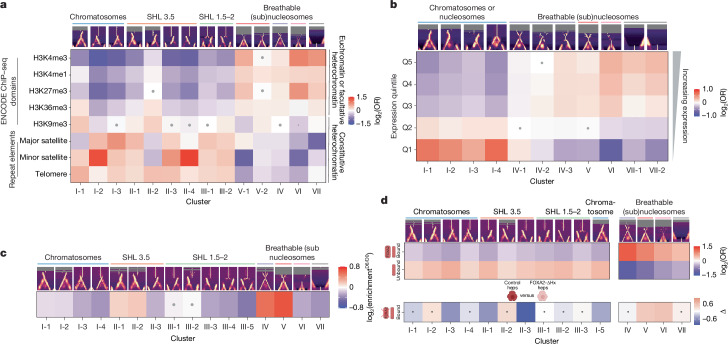
Nucleosome types show epigenome and promoter-strength specificity, are reprogrammed over developmental trajectories and are present in primary cells. **a**, Heat map depiction of enriched or depleted nucleosome types sampled from a wide variety of epigenomic domains or repeat types, from the E14 mouse ES cell dataset. The bottom rows represent heterochromatic repeat elements that do not fall within ENCODE ChIP–seq domains owing to repeat content, but that can be defined purely through sequence owing to the long-read nature of SAMOSA data. Grey dots represent insignificant Fisher’s exact test results (*q* > 0.1). **b**, Heat map depiction of enriched or depleted nucleosome types from a window surrounding the TSS of all annotated mouse genes (for the E14 mouse ES cell dataset), stratified by expression level in mouse ES cells. Grey dots represent insignificant Fisher’s exact test results (*q* > 0.1). **c**, Heat map of differential nucleosome-type enrichment for nucleosome types called at ChIP-backed FOXA2-binding sites for day 6 (D6) human endoderm and day 0 (D0) human iPS cells. **d**, Heat map of log_2_-transformed ORs for assessing the enrichment (red) or depletion (blue) of structurally distinct nucleosome types at bound versus randomly sampled FOXA2 motifs in C57BL/6J hepatocytes. ORs designated with a grey dot indicate those that are not statistically significant in a Fisher’s exact test (*q* > 0.1). The final row shows a heat map of effect sizes (Δ) for nucleosome types at bound FOXA2 motifs in heterozygous FOXA2-ΔHx hepatocytes (heps) versus wild-type hepatocytes (derived from age-matched littermate controls). Effect sizes designated with a grey dot indicate those that are not statistically significant in a Fisher’s exact test (*q* > 0.1). The illustrations of cell types in **d** were created in BioRender; Ramani, V. https://BioRender.com/ckmwvd1 (2026).

We hypothesized that nucleosome types reflect aspects of chromatin regulation. To test this, we again used enrichment tests, to quantify differences in nucleosome-type distribution across various epigenomic domains in E14 mouse ES cells (Fig. 5a). Notably, we found that distinct nucleosome types were significantly depleted or enriched across a wide range of both mappable epigenomic domains (defined by published histone modification ChIP–seq data) and repetitive genomic regions (typically unmappable by short-read assays). In predicted promoter regions (defined by H3K4me3), for instance, we observed significant enrichment for types VI and VII (VI OR = 1.856, *q* < 1.79 × 10^−308^; VII OR = 1.670, *q* = 1.16 × 10^−294^). In mouse minor satellite sequences, we observed a significant enrichment for clusters I-2 and II-4 (I-2 OR = 2.468, *q* < 1.79 × 10^−308^; II-4 OR = 2.559, *q* < 1.79 × 10^−308^). These analyses suggest that nucleosome types preferentially localize to specific types of euchromatin and heterochromatin.

We further hypothesized that nucleosome-type distributions vary with the promoter strength of Pol II-transcribed genes. We binned the mouse ES cell transcriptome into quintiles on the basis of transcriptional output, computed nucleosome types at annotated transcription start sites (TSSs) and computed their relative enrichment across quintiles (Fig. 5b). IDLI analysis again revealed similar nucleosome types to those above, with a greater representation of more accessible nucleosome types (groups IV–VII), consistent with our promoter-centric analysis. Enrichment or depletion of nucleosome types scaled with gene expression level (for example, significant enrichment of VI in quintile 5; VI OR in Q5 = 1.262, *q* = 4.62 × 10^−186^), implying that distinct nucleosome types either guide or reflect promoter activity.

**Extended Data Fig. 7:**
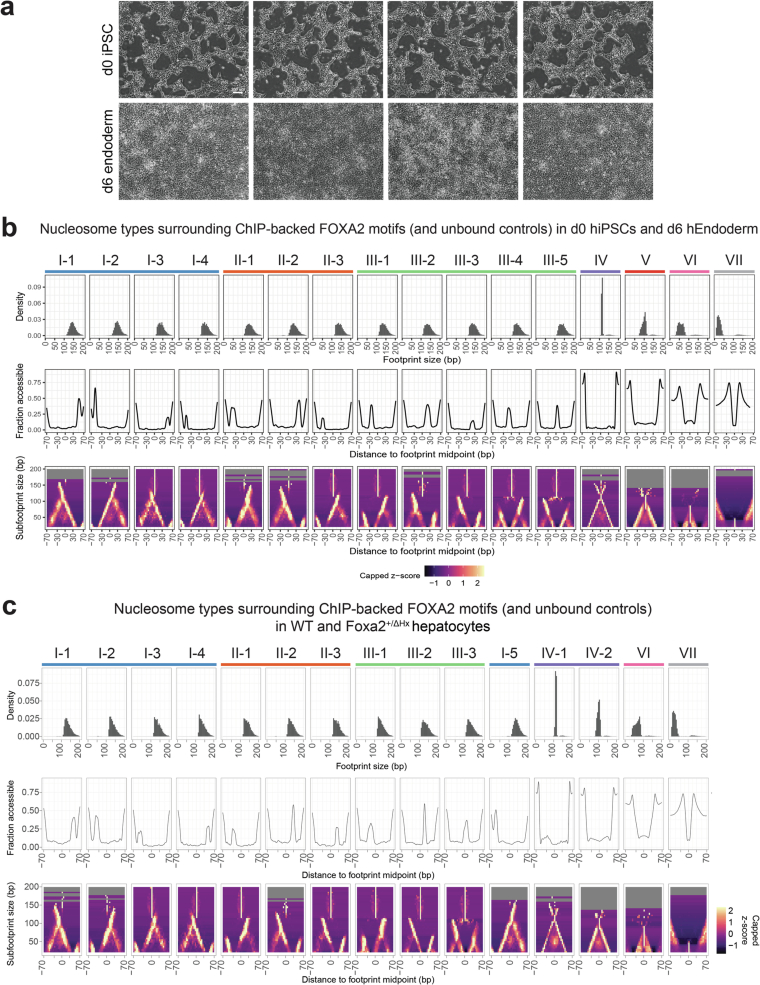
Differentiation images and nucleosome-type metadata for experiments in human iPS cells, human endoderm and mouse hepatocytes. **a**, Phase contrast micrographs of day 0 human iPS cells and day 6 differentiated human endoderm across four separate fields of view each. Scale bar, 200 µm. **b**, As above, nucleosome-type metadata from iPS cell and endoderm IDLI analyses, for footprints falling at FOXA2 motifs. **c**, As in **b** but for mouse hepatocyte data (including FOXA2-dHx hepatocytes).

Nucleosome landscapes are highly dynamic over developmental trajectories^61^. We thus sought to determine whether nucleosome types change during cellular differentiation in vitro, and in lineage-committed cells in vivo. We first performed SAMOSA experiments and IDLI analyses over in vitro differentiation, using human iPS cells differentiated into the endoderm germ layer (representative images in Extended Data Fig. 7a). IDLI analysis of footprinted day 0 (iPS cell) and day 6 (endoderm) samples, centred at bound FOXA2 motifs^62^, yielded 16 nucleosome types, again falling into 7 groups (Extended Data Fig. 7b). Enrichment analysis showed significant changes among these nucleosome types between undifferentiated iPS cells and differentiated endoderm (Fig. 5c). These included increased representation of nucleosomes with focal accessibility within the nucleosome core particle (II-1 OR = 1.106, *q* = 1.58 × 10^−05^), and breathable subnucleosomes (for example, V OR = 1.592, *q* = 9.49 × 10^−156^). These analyses suggest that nucleosome types are regulated at cell-type-specific, pioneer-factor-bound *cis*-regulatory elements during early development.

We next investigated whether regulated nucleosome types exist in vivo. We generated SAMOSA data in isolated hepatocytes for *n* = 7 mice, including a published genetic model^63^ heterozygous for a *Foxa2* allele lacking a crucial nucleosome-interacting domain (FOXA2-∆Hx). We then performed IDLI analyses on footprints drawn from regions surrounding ChIP-backed FOXA2-binding sites^64^ and matched negative controls, to assess nucleosome types in primary cells. Clustering yielded 15 nucleosome types falling into 7 groups (Extended Data Fig. 7c), suggesting that nucleosome distortion occurs in a terminally differentiated primary mammalian cell type. Computing the relative enrichment of nucleosome types at FOXA2-binding sites compared with random motif matches (Fig. 5d), we observed a similar TF-dependent pattern to that seen for SOX2 in mouse ES cells, with breathable (sub)nucleosomes and short footprints significantly enriched (for example, IV-1 OR = 2.284, *q* = 2.16 × 10^−68^), and inaccessible nucleosomes significantly depleted. Differential nucleosome-type analysis in FOXA2-∆Hx heterozygous hepatocytes versus wild-type littermate controls revealed significant depletion of two nucleosome types: II-3 (mutant OR = 0.624, *q* = 6.45 × 10^−11^) and I-5 (mutant OR = 0.820, *q* = 8.02 × 10^−4^). We conclude that nucleosome distortion occurs at cell-type-specific regulatory elements in vivo, and that a histone-binding domain of FOXA2 directly contributes to nucleosome distortion at associated binding sites.

## Single-molecule co-occurrence of nucleosome types

**Extended Data Fig. 8:**
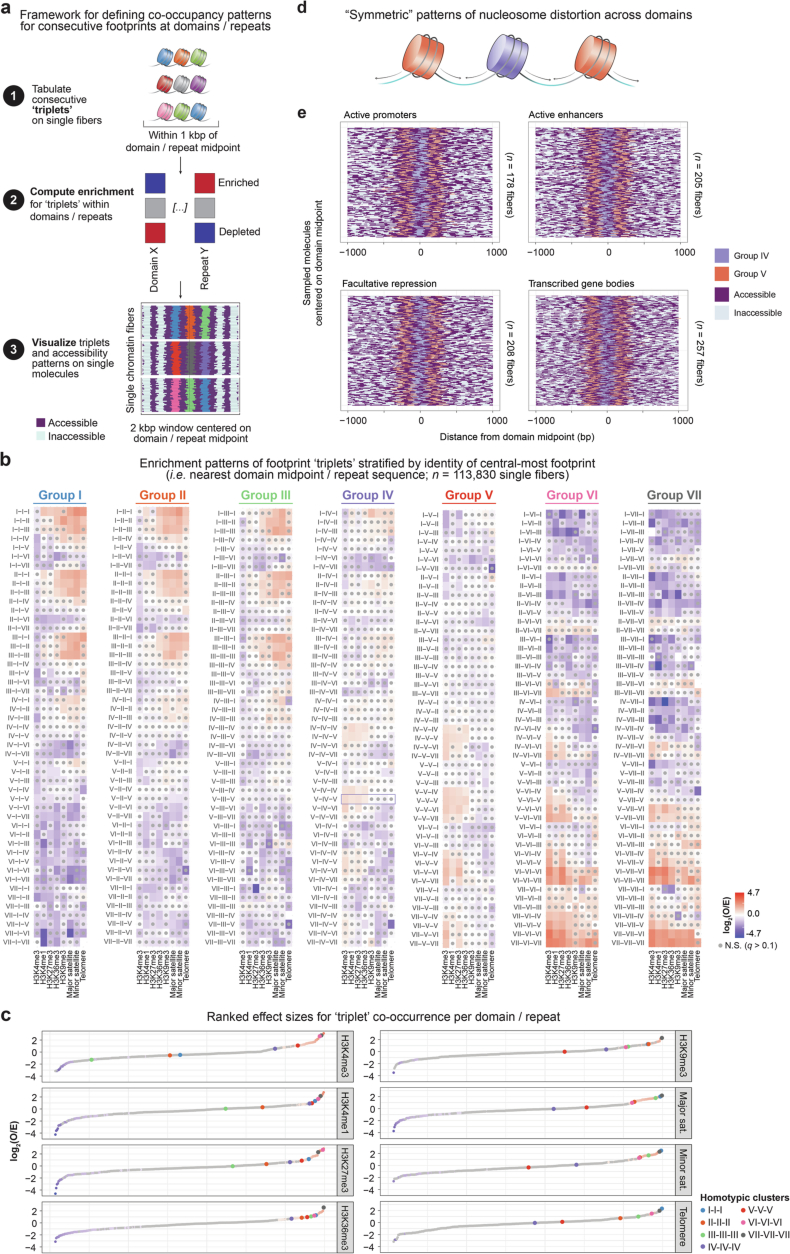
Co-occupancy analysis reveals enrichment of homotypic clusters of footprints with shared nucleosomal distortion patterns on individual fibres. **a**, Domain- and repeat-associated footprints were analysed for co-occupancy patterns on single chromatin fibres. Instances of three consecutive footprints on individual sequencing reads nearest domain or repeat midpoints were tabulated based on their associated group assignments. **b**, Heat map of log_2_-transformed O/E ratios computed for each possible ‘triplet’ on a per-domain or per-repeat basis. Effect sizes designated with a grey dot indicate those from Fisher’s exact test that are not statistically significant (Storey *q*-value > 0.1). **c**, Waterfall plots of ranked effect sizes stratified by locus indicates enrichment of homotypic clusters of footprints with shared nucleosomal distortion patterns. As in **b**, grey dots indicate effect sizes for ‘triplets’ with *q* > 0.1. Red and blue dots indicate ‘triplets’ that are enriched and depleted within a given locus, respectively. ‘Triplets’ that comprise homotypic clusters are highlighted by larger coloured dots (see legend on bottom right). **d**, Schematic for ‘triplets’ of symmetric pattern of ‘breathable’ nucleosomes (IV-V-IV), which are statistically enriched in active and facultatively repressed chromatin. **e**, Single-molecule visualization of chromatin fibres with IV-V-IV ‘triplets’, in which each row represents a unique sequenced fibre.

A key advantage of single-molecule footprinting approaches is their unique ability to measure the co-occurrence of protein–DNA interactions^65,66^. Owing to the multi-kilobase length scale of SAMOSA footprinting data, IDLI can assess the statistically enriched co-occurrence of nucleosome types on individual fibres. We implemented an analysis pipeline (Extended Data Fig. 8a) to compute whether types of trinucleosome stretches co-occur more frequently than expected by chance. We visualized test results in two ways: first, as a heat map of effect sizes (Extended Data Fig. 8b), and second, as waterfall plots of these effect sizes (Extended Data Fig. 8c). Our analysis revealed striking and significant patterns of co-occupancy of nucleosome types (binned by ‘group’ for this analysis), including an enrichment of ‘homotypic’ arrangements specifically in active versus repressed epigenomic domains (for example, significant enrichment of VI-VI-VI trinucleosomes in H3K4me1 chromatin; OR = 3.189, *q* = 4.48 × 10^−3^). Our analysis also uncovered triplet arrangements that were found to be enriched on both active and facultatively repressed chromatin, including a symmetric pattern of IV-V-IV (cartoon shown in Extended Data Fig. 8d). Single-molecule visualization of fibres falling within each of four different chromatin types (Extended Data Fig. 8e) highlights the flexibility of these analyses, while illustrating trinucleosomal co-occurrence motifs at single-molecule resolution and at any sequence of interest genome-wide. Together, these analyses reveal epigenome-specific co-occurrence patterns of distinctly structured nucleosomes.

## Discussion

**Extended Data Fig. 9:**
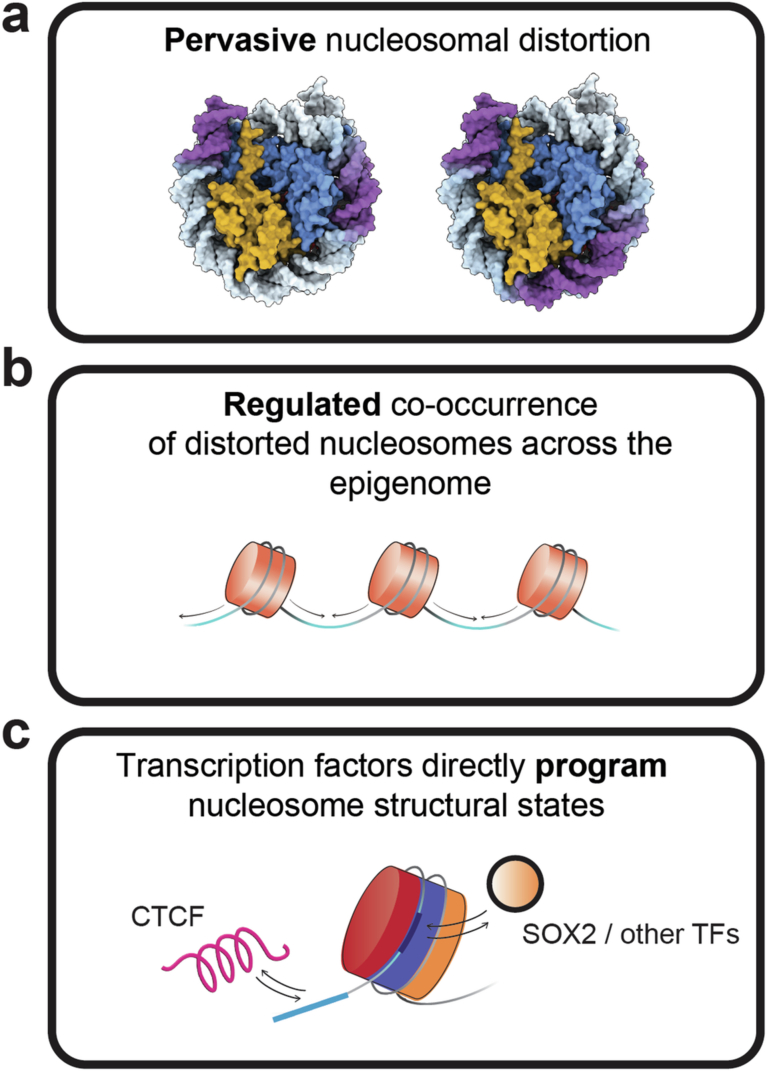
Visual summary of findings. **a**, IDLI reveals that nucleosomes in cells (nucleosome types) display varying degrees of DNA accessibility along the nucleosomal wrap. **b**, Clustering and subsequent analysis reveals widespread nucleosome distortion patterns across length scales ranging from individual chromatin fibres, to promoters of different strengths, to large-scale epigenomic domains such as repetitive heterochromatic loci. **c**, We discover that TFs directly relate nucleosome distortion. Specific nucleosome types contain specific TF motifs, and direct depletion of TFs demonstrates a causal regulatory role for the TFs CTCF and SOX2.

We report the discovery of pervasive, patterned and programmed nucleosome distortion across mammalian epigenomes (Extended Data Fig. 9). Using a newly developed analytical pipeline (IDLI) we show that nucleosomes across diverse epigenomic states exist predominantly in a partially accessible state (Extended Data Fig. 9a). The extent of this accessibility ranges from focal points at specific sites along the nucleosomal wrap, to symmetrically unwound and breathable nucleosomes and subnucleosomes. Our pipeline also classifies short DNA protections; given the raw abundance of these protections, these are most likely to be a result of the histone H2A–H2B dimer, although our analyses at TF-binding sites show a quantifiable (and expected) contribution of TFs to the overall number of short protections. Given the increasing evidence that chromatin structure in cells is highly dynamic, we speculate that types defined here reflect the intrinsic mobility of nucleosomes in cells.

IDLI analyses capture regulated patterns of nucleosome distortion genome-wide (Extended Data Fig. 9b). We validate these patterns using integrative genomic, genetic and biochemical analyses. Our proof-of-concept study shows that nucleosome types—which we interpret as structurally distinct nucleosomes—are specifically enriched and depleted across epigenomic domains and TSSs of varying strengths; furthermore, our single-molecule co-occupancy analyses reveal a statistically significant co-occurrence of structurally distinct nucleosomes on individual chromatin fibres. Future work must assess the significance of these co-occurrence patterns at even higher resolution, and in the context of a larger set of TF-binding motifs. Given the short residence times of most TFs^67^, however, we stress that a comprehensive, TF-specific co-accessibility analysis will require SAMOSA data with increased genomic coverage specifically at target TF-binding sites. This may be achieved through additional technology improvements (for example, higher-throughput multiplex target capture of TF-binding sites^68^ (analogous to multiplex bisulfite sequencing^66,69^) and targeted deaminase footprinting^70,71^).

Finally, we show that TFs directly program nucleosome distortion at cognate binding sites (Extended Data Fig. 9c). Distinct nucleosome types are associated with specific TF motif families (for example, homeodomain motifs, E-boxes and C2H2 ZF motifs). Using small-molecule-sensitive degrons (CTCF and SOX2) and a mutant *Foxa2* allele defective for histone binding, we provide evidence that TFs directly influence nucleosome structure in cell culture and in vivo. Our results for SOX2 suggest that there are multiple modes in which TFs interact with DNA motifs and/or nucleosomes. SOX2 depletion results in a genome-wide increase in accessibility at unbound motifs and, paradoxically, results in a loss of less-accessible nucleosome types, indicating that SOX2 has a direct role in binding and stabilizing nucleosomes. We speculate that this effect stems from the innate ability of factors such as SOX2 (ref. ^20^) and HMGB1 (ref. ^46^) to engage specific points along the nucleosome wrap, such as SHL ±2. We observe similar patterns at FOXA2 motifs in primary mouse hepatocytes. FOXA2 exists within a wider family of FOXA paralogues that together are essential for liver function^72^. Compared with unbound control motifs, FOXA2-regulated sites are specifically enriched for accessible (sub)nucleosomes and short protections, supporting previous models for a motif-encoded grammar for these interactions^73^. Altering the dosage of intact FOXA2 protein by replacing one allele with FOXA2-∆Hx has a significant effect on the observed distribution of nucleosome types at FOXA2-binding sites, resulting in a loss of nucleosomes with accessibility at SHL ±3.5, as well as chromatosomes. Because homozygous FOXA2-∆Hx is embryonic lethal^63^, these findings also suggest that IDLI is sensitive enough to detect altered pioneer TF interactions with the nucleosome, even in the presence of a functional copy of the protein in the nucleus.

Looking ahead, we expect IDLI to be applicable to a wide variety of problems in chromatin biology. ATP-dependent chromatin remodelling^3,74^, nucleosome assembly, transcription^75^, replication^52^ and all other nuclear processes regularly generate structurally distorted nucleosomes, but prevailing approaches to study this distortion rely on in vitro biochemical and biophysical assays. IDLI promises to bridge the gap between highly resolved biochemistry and in-nucleus chromatin structure through genome-wide and near-nucleotide-precise maps of structurally distorted states in vivo. Combining bottom-up biochemistry, the top-down structural classification presented here and approaches such as in situ chromatin imaging^29^ will enable deep quantitative insight into the in vivo biochemistry of essential nuclear processes.

## Methods

### SAMOSA from E14 mouse ES cells

We compiled a large compendium of SAMOSA data from wild-type E14 mouse ES cells, comprising both newly generated datasets and published datasets from our laboratory, including: (1) our study on the function of ISWI-family remodelling complexes^3^; (2) samples prepared with our SMRT-Tag protocol, which uses a transposase-based strategy for PacBio library preparation from lower input amounts of footprinted DNA^38^; and (3) mouse ES cells labelled with the halogenated thymidine analogue BrdU for varying time points in our study of single-fibre accessibility patterns in newly replicated chromatin^52^. Only samples with a mononucleosome–dinucleosome ratio (MDR; calculation detailed in ‘Data analysis and visualization’) greater than 10 were included for analysis. All cell lines were routinely tested for mycoplasma contamination. No statistical models were used to predetermine sample size. Neither blinding nor randomization was used.

#### Cell culture

For newly generated libraries, E14 mouse ES cells were grown on gelatin-coated (0.1% solution in 1× PBS) tissue culture plates. Cells were cultured in mouse ES cell medium (DMEM with GlutaMAX (Thermo Fisher Scientific, 10566-016)), supplemented with 15% fetal bovine serum (FBS; Thermo Fisher Scientific, SH30071.03), 14.2 mM 2-mercaptoethanol (Bio-Rad, 1610710), 1× NEAA (Thermo Fisher Scientific, 11140-50), 1 mM sodium pyruvate (Thermo Fisher Scientific, 11360-070) and 1× LIF (purified by the laboratory of B. Panning). Some samples were cultured with MEK (1 µM PD0325901 (Apex Bio/Fischer, A3013-25)) and GSK3β (3 µM CHIR99021 (Apex Bio/Fischer, A3011-100)) inhibitors (2i). The medium was changed daily and cells were passaged with 1× TrypLE (Thermo Fisher Scientific, 12605010) when confluent. To collect mouse ES cells, cells were rinsed with 1× PBS (Thermo Fisher Scientific, 10010023), dissociated with 1× TrypLE, quenched with mouse ES cell medium and centrifuged at 500*g* for 5 min. All mouse ES cell lines used in this study were regularly tested for mycoplasma contamination (Lonza LT07-318).

#### MTase footprinting and library preparation

Live cell counts were estimated (Countess 3) to ensure equal numbers of nuclei per unit MTase were used in each footprinting reaction. Nuclei were isolated by incubating 1 × 10^6^ live mouse ES cells in 1 ml ice-cold NE1 buffer (20 mM HEPES, 10 mM KCl, 1 mM MgCl_2_, 0.1% Triton X-100, 20% glycerol and 1× protease inhibitor (Roche, 4693132001)) on ice for 5 min. Nuclei were pelleted by centrifugation at 500*g* for 5 min at 4 °C. Nuclei were rinsed in 1 ml buffer M (15 mM Tris-HCl pH 8.0, 15 mM NaCl, 60 mM KCl and 0.5 mM spermidine) and re-pelleted by centrifugation at 500*g* for 5 min at 4 °C. For footprinting, nuclei were resuspended in 200 µl buffer M with 1 mM SAM (NEB, B9003S) and 10 µl high-concentration EcoGII (custom order from NEB). Nuclei were incubated at 37 °C for 30 min, with a spike-in of 1 µl 32 mM SAM stock halfway through the footprinting reaction. For light MNase digest to liberate chromatin fragments, footprinted nuclei were pelleted by centrifugation at 500*g* for 5 min at 4 °C, resuspended in 200 µl buffer M with 1 mM CaCl_2_ and 0.02 U MNase (Sigma N3755) and incubated on ice for 30 min. To quench the MNase reaction, 2 mM EGTA was added.

To purify DNA, samples were treated with 10 µl RNaseA (Thermo Fisher Scientific, AM2270) for 10 min at 37 °C, followed by the addition of 2 µl 10% SDS and 2 µl 20 mg ml^−1^ proteinase K (Thermo Fisher Scientific, AM2548) for more than two hours (up to overnight) at 65 °C. To extract DNA, an equal volume (around 250 µl) phenol-chloroform-isoamyl alcohol was added, and samples were mixed by brief vortexing and centrifuged at maximum speed for 2 min. The aqueous phase was transferred to a fresh tube along with 0.1 volumes 3 M NaOAc, 2 volumes ice-cold 100% ethanol and 1 µl GlycoBlue co-precipitant (Thermo Fisher Scientific, AM9516). Samples were mixed by inverting tubes and incubated overnight at −20 °C, centrifuged at maximum speed for 30 min at 4 °C, washed in 500 µl ice-cold 70% ethanol, air dried and resuspended in 30 µl buffer EB. DNA concentrations were measured using the Qubit dsDNA High Sensitivity Quantification Kit (Thermo Fisher Scientific, Q32851). Up to 1 µg DNA was prepared into PacBio SMRT libraries with the SMRTbell Express Template Prep Kit 2.0 as per the manufacturer’s directions. Fragment size distributions were estimated by Femto Pulse (Agilent). Libraries were sequenced on PacBio Sequel II 8M SMRT cells.

### SAMOSA from SOX2-FKBP mouse ES cells

Degron-tagged *Sox2*^*FKBP-F36V*^ (hereafter referred to as SOX2-FKBP) mouse ES cells were provided by the laboratory of E. de Wit^60^. SOX2-FKBP mouse ES cells were cultured in the same mouse ES cell medium described above (including 2i) and were plated at a density of 5 × 10^5^ per well in 6-well plates 24 h before collection. For degradation, SOX2-FKBP mouse ES cells were treated with 0.5 µM dTAG^V^-1 (Tocris 6914) for varying time points (30 min, 2 h or 6 h). As a control, SOX2-FKBP mouse ES cell samples were treated with 0.5 µM dTAG^V^-1-NEG (Tocris 6915) for six hours before collection. All experiments were performed in biological duplicate. Footprinting and downstream steps were performed as detailed in the section above, except that instead of the MNase digest step, we sheared purified genomic DNA with Covaris g-TUBEs (Covaris, 520079; 4,600*g* for six passes).

#### Western blot for SOX2 protein in nuclear extracts

To validate levels of SOX2 knockdown, mouse ES cell nuclei were isolated in NE1 buffer, as was done for the matched MTase-footprinted samples. Pelleted nuclei were resuspended in 100 µl ice-cold RIPA buffer (150 mM NaCl, 1% NP-40, 0.5% sodium deoxycholate, 0.1% SDS, 50 mM Tris pH 7.4 and 1× protease inhibitor (Roche 4693132001)). Samples were incubated for 30 min on ice and sonicated briefly to solubilize chromatin. Protein extracts were clarified by centrifugation at 12,000*g* for 20 min at 4 °C. Soluble material was collected, protein concentrations were measured by bicinchoninic acid assay (Thermo Fisher Scientific, 23227), and samples were diluted in NuPAGE LDS sample buffer (Thermo Fisher Scientific, NP0007) with 5% β-mercaptoethanol. Samples were reduced by boiling for 10 min at 95 °C, resolved on 4–12% Bis-Tris gels (Thermo Fisher Scientific, NP0322), and transferred to PVDF membranes. Membranes were incubated overnight with the following primary antibodies: anti-SOX2 (CST 23064S; 1:1,000 dilution) and anti-vinculin (Sigma V9264; 1:1,000 dilution; used as loading control). After washing, membranes were incubated with secondary antibodies conjugated to IRDye 700 or IRDye 800 (1:10,000 dilution) and imaged with the LiCor Odyssey.

### SAMOSA from CTCF-AID mouse ES cells

CTCF-AID mouse ES cells (EN52.9.1) were generated by the E.N. laboratory^76^. Raw data from CTCF-AID samples used in this study were previously published by our group^52^, and samples were required to have a MDR (calculation detailed in ‘Data analysis and visualization’) greater than 10 to be included in analyses. These criteria resulted in: (1) *n* = 10 biological replicates of auxin-treated (for six hours) CTCF-AID mouse ES cells; and (2) *n* = 6 biological replicates of ddH_2_O-treated CTCF-AID mouse ES cells (as controls).

### SAMOSA from NIPBL-FKBP mouse ES cells

NIPBL-FKBP mouse ES cells (EA18.1) were cultured in the same conditions as E14 mouse ES cells and NIPBL was depleted using 500 nM PROTAC dTAG-13 (Sigma-Aldrich, SML2601), as described previously^55,56^. EA18.1 cells were treated with dimethyl sulfoxide (DMSO) for 24 h, dTAG-13 for 6 h (preceded by DMSO) or dTAG-13 for 24 h, with *n* = 2 biological replicates per condition. Cells were also pulsed with BrdU for one hour before collection. SAMOSA was done as for wild-type E14 mouse ES cells, with no MNase treatment; instead, DNA was sheared using g-TUBEs (Covaris 520079) to a target length of 7–8 kb. Replicates were pooled and the 6-h and 24-h time points were combined for treatment conditions to cover the full range of phenotypes that are relevant to perturbing loop extrusion.

### SAMOSA from primary mouse hepatocytes

#### Mouse husbandry and genotyping

All animal experiments were performed under the supervision and approval of the Institutional Animal Care and Use Committee (IACUC) at the University of California, San Francisco (UCSF) (protocol AN179718-03F). Six-week-old, C57BL/6J mice (Jackson Laboratory, 000664) were used as wild-type hepatocyte samples in our dataset (*n* = 3 biological replicates). *Foxa2*^∆Hx-*TagRFP*^-expressing mice (hereafter referred to as FOXA2-ΔHx) were provided by the laboratory of K. Zaret^63^. Samples in this study included heterozygous FOXA2-ΔHx mice (*n* = 3; one male and two females) and a littermate wild-type control male mouse.

Genotyping for the presence (around 230 bp) or absence (around 200 bp) of the associated Tag-RFP fluorophore was performed with the following primer pair:

lox2272_F: AGTGTTGTCTTCTGCCTTTGAG

lox2272_R: GCTTACCTTAGTCTCGGTCTTGG

Genotyping for the presence (around 300 bp) or absence (around 270 bp) of the helical domain in FOXA2 was performed with the following primer pair:

TagRFP_F: GCTCTTCGCCCTTAGACACC

TagRFP_R: ATCAGCCCCACAAAATGGAC

#### Isolation of primary mouse hepatocytes

Hepatocyte isolation was performed by the Liver Cell Isolation, Analysis and Immunology Core at the UCSF Liver Center. After flushing the liver free of blood, the organ was perfused for 3 min at 4 ml per min with Liver Perfusion Medium (Thermo Fisher Scientific, 17701038), followed by an additional 7 min at 4 ml per min with Liver Digest Medium (Thermo Fisher Scientific, 17703034). The liver was then removed, diced with scissors and suspended in DME-H21 with insulin-transferrin-selenium (Thermo Fisher Scientific, 41400045), penicillin–streptomycin and 5% FBS. The crude suspension was strained through a 100-µm filter and pelleted twice at 60*g* with interval washing. Hepatocytes were purified by suspension of the pellet and centrifugation through 45% Percoll (Cytiva 17089102) at 130*g* for 15 min. Purified hepatocytes, which pelleted through the Percoll, were suspended in DME-H21 as above. Final hepatocyte suspensions had a viability higher than 90%.

#### MTase footprinting for hepatocyte samples

Footprinting reaction for samples denoted HepWT_Rep1 and HepWT_Rep2 was performed in 1 × 10^6^ purified hepatocyte nuclei per library. Footprinting and downstream steps were performed as described above, except that primary hepatocytes were first dounced in NE1 buffer (10× with loose pestle and 10× with tight pestle) to facilitate nuclear isolation.

The footprinting reaction for all remaining hepatocyte samples was performed with 1 × 10^6^ digitonin-permeabilized hepatocytes. Footprinting and downstream steps were performed as described above, with two notable exceptions. First, instead of isolating nuclei with NE1 buffer, we directly permeabilized cells by adding 0.05% digitonin (Thermo Fisher Scientific, BN2006) to buffer M when performing the EcoGII footprinting reaction. Second, instead of treating nuclei with MNase to liberate chromatin fragments, we sonicated purified genomic DNA with the Megaruptor 3 (concentration, 10 ng µl^−1^; volume, 100 µl; speed, 031) to achieve more consistently sized and longer molecules (>10 kb).

### SAMOSA from H1 TKO mouse ES cells

ES cell lines derived from H1c/H1d/H1e TKO and wild-type littermates were expanded on mitotically inactivated mouse embryonic fibroblasts (MEF) feeder layers as we described previously^77^. Before extraction of nuclei, MEF feeder cells were depleted from ES cell culture and H1 TKO and wild-type ES cells were subsequently cultured feeder-free on 0.1% gelatin-coated tissue culture plates with 2i/LIF medium for three passages following protocols as described^78^. SAMOSA was performed on 1 × 10^6^ nuclei per replicate, as with E14 mouse ES cells, without MNase treatment. DNA was then purified and used directly for PacBio library preparation and sequencing.

### SAMOSA from iPS cells and differentiated endoderm

The normal human male iPS cell line GM25256 (WTC, hPSCreg: UCSFi001-A, generated at Gladstone Institutes, distributed by the Coriell Institute for Medical Research) was used for endoderm differentiation^79^. Undifferentiated iPS cells were cultured in mTeSR Plus medium (100-0276, STEMCELL Technologies) in six-well plates coated with 0.25 mg ml^−1^ Matrigel GFR basement membrane matrix (356231, Corning) at 37 °C in a humidified incubator with 5% CO_2_ and 5% O_2_. Cells were passaged every three days using Versene (A4239101, Gibco) and replated in mTeSR Plus medium supplemented with 10 µM Y-27632 (101763-964, Selleck Chemicals). Mycoplasma testing was performed every six months during routine culture using the MycoAlert Mycoplasma Detection Kit (LT07-218, Lonza).

Endoderm was differentiated from iPS cells using a modified version of a previously described protocol^80^. In brief, iPS cells were dissociated with Accutase (07920, STEMCELL Technologies) at 70–80% confluency and replated at a density of 75,000 cells per cm^2^ on Matrigel-coated six-well plates in mTeSR Plus medium including 10 µM Y-27632. After 18 h, cells were exposed to endoderm-induction medium, consisting of RPMI 1640 (11875093, Gibco) supplemented with 2% B-27 supplement minus insulin (A1895601, Gibco), 1% Glutamax (35050061, Gibco), 1% non-essential amino acid solution (NEAA; 11140050, Gibco), 0.5 mM sodium butyrate (B5887, Sigma-Aldrich) and 100 ng ml^−1^ activin A (120-14E, PeproTech). Cells were cultured at 20% O_2_ for six days. During the first three days of differentiation, the following compounds were added to endoderm-induction medium: 3 µM CHIR99021 (501742210, Sigma-Aldrich) on day 1; 10 ng ml^−1^ BMP4 (120-05ET, PeproTech) and 20 ng ml^−1^ basic fibroblast growth factor (FGFb; 100-18B, PeproTech) on day 1 and day 2; 50 nM PI103 (501932056, Thermo Fisher Scientific) from day 1 to day 3; and knockout serum replacement (KSR; 10828010, Gibco) at the following concentrations: 2% on day 1, 1% on day 2 and 0.5% on day 3. The medium was changed every day during differentiation and maintenance.

SAMOSA was performed on approximately 1 × 10^6^ digitonin-permeabilized nuclei per replicate, for both iPS cells and endoderm. DNA was purified then sheared using the Megaruptor 3 to a target length of 15 kb before PacBio library preparation and sequencing.

### SAMOSA-ChAAT on reconstituted Widom arrays

Data from HMGB1 and linker histone H1 Widom 601 array SAMOSA-ChAAT experiments performed in and described in detail in a previous study^46^ were reprocessed using the IDLI pipeline as below.

### Preprocessing PacBio data for single-molecule accessibility

To preprocess PacBio data, we used software from Pacific Biosciences and a custom script (hmm_output_t_values.py) to successively run the NN-HMM across a range of user-specified transition probabilities. These resulted in the following output per sample: (1) an alignment of CCS reads to the mouse reference genome (mm10); (2) an accessibility prediction per CCS molecule (Viterbi path of HMM component of model); and (3) an atlas of footprint locations (start/end positions) and sizes on a per-molecule basis. Files for (2) and (3) were generated for *t* = 1/1,000, 31/1,000, 51/1,000, 71/1,000 and 101/1,000 for each sequencing library.

### Data analysis and visualization

#### Quantifying the extent of MTase footprinting per sequencing library

We used the ratio of mononucleosome-sized to dinucleosome-sized footprints (MDR) as a proxy for measuring the extent of EcoGII footprinting on a per-sample basis. Poorly methylated samples exhibit a higher fraction of footprints longer than the expected length of DNA protected by a mononucleosome (lower MDR values), which can be ascribed to the failure of EcoGII to efficiently methylate in linker DNA. We used a custom script (compute_mdr_per_sample.py), which uses the find_peaks function from scipy and computes the ratio of maximum peak heights in footprint length histograms for mononucleosome- and dinucleosome-sized footprints per sequencing library.

#### Visualizing single-molecule data across different *t* values at single genomic loci

To visualize SAMOSA data at loci of interest, data were processed with a custom script (generate_bam_for_igv.py) to encode single-molecule accessibility patterns (for any given *t* value) as an additional flag in aligned BAM files. These BAM files were concatenated across E14 mouse ES cell samples with MDR > 10 and visualized at the *Sox2* locus (chr. 3: 34,756,929–34,759,161; including the *Sox2* promoter, gene body and downstream SCR) using a custom version of IGV (https://github.com/RamaniLab/SMRT-Tag/tree/main/igv-vis). Bulk ATAC-seq data from mouse ES cells were obtained as processed BW files (GSE98390) and visualized at identical coordinates on the UCSC Genome Browser.

#### Identifying fibres that overlap annotated epigenomic domains in mouse ES cells

A custom script from our group (https://github.com/RamaniLab/SAMOSA-ChAAT/blob/main/scripts/zmw_selector.py) was adapted to identify sequencing reads in which a portion of the read falls within ±1 kb of midpoints of histone post-translational modification-defined epigenomic domains in mouse ES cells (H3K4me1, H3K4me3, H3K9me3, H3K27me3, and H3K36me3 ChIP–seq peaks; derived from ENCODE data).

#### Identifying fibres that overlap with repeat elements in the mouse genome

A custom script from our group (https://github.com/RamaniLab/SAMOSA-ChAAT/blob/main/scripts/blast_bam.sh) was used to identify sequencing reads with one or more matches to repeat elements of interest. In brief, we ran BLAST on CCS reads from a database consisting of mouse major satellite sequence (M32564.1), minor satellite sequence (X14462.1) and telomeric DNA sequence (pentameric repeats of TTAGGG). For fibres with multiple repeat matches, only the repeat sequence with the lowest E-value was considered for downstream analyses.

#### Identifying fibres that overlap with bound and unbound TF motif matches

To define instances of TF-bound motifs, we referenced the following datasets: ChIP-nexus data for SOX2 in mouse ES cells (GSE137193)^59^, ChIP–seq data for CTCF in mouse ES cells (ENCFF508CKL) and ChIP–seq data for FOXA2 in mouse hepatocytes (GSE157452)^64^. For SOX2 motifs, we retained only the subset of peaks that contained a SOX2-OCT4 composite motif match as defined by this dataset^81^. For CTCF data, we used a curated set of ChIP–seq backed motifs first processed in this study^82^. For FOXA2 data, we retained only the subset of peaks that contained a position weight matrix (PWM) match (MA0047.2) for the FOXA2 motif^83^. For control sites, we randomly sampled an equal number of motif matches per factor of interest from a previously published list of motifs across the mouse genome^82^, requiring that these motifs fall at least 1 kb away from the nearest ChIP-annotated peak. As above with epigenomic domains, we used a custom script to identify sequencing reads in which a portion of the read falls within ±1 kb of motif midpoints.

#### Clustering single-molecule accessibility patterns with respect to footprint midpoints

To examine patterns of nucleosomal distortion, we clustered accessibility patterns across a range of *t* values with respect to footprint midpoints. Footprints less than 200 nt in length (intended to capture both nucleosomal and subnucleosomal species) within ±500 bp of midpoints of epigenomic domains, repeat sequences or TF-binding motifs were selected. On a per-footprint basis, accessibility data (from *t* = 31/1,000, 51/1,000 and 71/1,000) for a 140-bp window centred on each footprint midpoint (420 bp in total per footprint) were used as input for Leiden clustering (res = 0.5, 0.6, 0.6 and 0.6 for domains/repeats, SOX2, CTCF and FOXA2 motifs, respectively). Clusters were filtered such that the least abundant clusters that collectively summed up to 10% of each dataset were removed. It should be noted that: (1) accessibility data were oriented accounting for sequence feature strand before clustering to preserve any potential directional effects associated with non-palindromic TF-binding motifs; (2) this approach was intended to enable clustering on accessibility information across multiple *t* values per footprint; and (3) this window size was selected to prioritize signal from SHL −7 to SHL +7 for nucleosomal footprints. Representative code for performing this analysis is provided as a custom script (process_footprints_nucleosomal_distortion.py).

#### Plotting footprint size distributions, mean accessibility patterns and horizon plots per cluster

To assign putative biological functions to clusters of nucleosomal distortion patterns or translational positions, we visualized the following features. First, we plotted the distribution of footprint sizes on a per-cluster basis for data from the lowest *t* value examined (*t* = 1/1,000). Second, we plotted mean accessibility as a function of distance to respective footprint/sequence feature midpoints from the highest *t* value used for Leiden clustering (*t* = 71/1,000). Third, we generated horizon plots (analogous to commonly used V-plots in MNase-seq data) by computing z-scores for the enrichment of footprints of a particular size (20–200 nt) at specific positions (±70 bp from footprint/sequence feature midpoints) for each individual cluster. *z*-scores are summed per size–position combination across the range of *t* values previously used for clustering (*t* = 31/1,000, 51/1,000 and 71/1,000). Colour scale limits for the resulting heat map are capped by the 0th (lower) and 95th (upper) percentile z-scores for positions within ±70 bp of the footprint/sequence feature midpoint. Clusters of entirely unmethylated footprints were manually filtered out before downstream quantification. Representative code for performing this analysis is provided as a custom script (plot_footlen_meanacc_horizondata.py).

#### Identifying groups of clusters with shared nucleosomal distortion patterns or translational positions

To group Leiden assignments for nucleosomal distortion patterns or translational positions in an unbiased manner, we performed hierarchical clustering of horizon plot data as follows. *z*-scores per cluster were filtered such that only those corresponding to footprints less than 100 nt and less than 50 nt in length were retained for defining nucleosomal distortion and translational position groups, respectively. This choice was intended to prioritize the clustering of horizon plot data specifically within subfootprint size ranges that capture subnucleosomal organization. Data were manually inspected and *z*-scores flipped with respect to footprint/sequence feature midpoints to account for the nucleosomal dyad as an axis of symmetry. The distance matrix was computed with the dist function in R, hierarchical clustering was performed with hclust (from the factoextra package; method = ward.D2) and output was visualized as a dendrogram (with fviz_dend). In most cases, the group definitions are driven by the dendrogram. In a subset of cases, we found that footprint size drove the clustering, but patterns were visually most similar to alternative groups. In those cases, we manually assigned a label.

#### UMAP visualization of footprint types within epigenomic domains and at repeat sequences

From accessibility data for footprints within histone-modification-defined domains and at mouse repeat elements, we used Scanpy (v.1.9.3) for principal component analysis (PCA)-based dimensionality reduction, construction of a *k*-nearest neighbours graph (metric = correlation, *n*_neighbours = 15) and UMAP visualization (min_dist = 0.5). Clusters were coloured according to Leiden assignments and proportions of each Leiden-defined cluster were represented as a pie chart surrounding the UMAP visualization.

#### Structural visualization of nucleosomal accessibility patterns

For structural visualization of mean accessibility patterns from defined clusters, we used a custom script (accessibility_to_pdb_structure.py) to convert our mean single-molecule accessibility data per cluster of interest from the highest *t* value used for Leiden clustering (*t* = 71/1,000) into a format compatible with visualization via ChimeraX (v.1.7.1; attribute = percentAcc, match mode = 1-to-1 and recipient = residues)^84^. Accessibility values were overlaid on Protein Data Bank (PDB) structure 7KBD (ref. ^44^).

#### Computing the enrichment of nucleosomal distortion patterns and translational positions across distinct genomic loci

To compute ORs for Leiden-defined clusters across epigenomic domains and repeat elements, or for bound versus randomly sampled TF motifs, Fisher’s exact tests were done with scipy (in Python). All computed *P* values were corrected with a Storey *q*-value correction, using the qvalue package in R.

#### Computing effect sizes associated with TF knockdown or across genotypes

To compute effect sizes after degron-mediated TF knockdown or across mouse genotypes, Fisher’s exact tests were performed for footprints specifically at TF-bound motifs (for example, footprints from dTAG-^V^1 versus DMSO-treated SOX2-FKBP mouse ES cells) with scipy (in Python). All computed *P *values were corrected with a Storey *q*-value correction, using the qvalue package in R.

#### Reproducibility of nucleosomal distortion patterns or translational positions across replicates

To assess reproducibility in our large-scale E14 mouse ES cell dataset, we asked whether the rank order of clusters of nucleosomal distortion patterns or translational positions is preserved across sequencing libraries. To do this, the relative proportion of each Leiden-defined cluster at domains/repeats or at TF-bound motifs was computed on a per-sample basis and analysed using Spearman’s correlation (with spearmanr from scipy). Footprints from all sequencing libraries with MDR > 10 were included in Leiden clustering of accessibility patterns; this choice was intended to include as many footprints as possible to increase statistical power. However, for these correlation analyses, we imposed a minimum sequencing depth for a biological replicate to be included, with the goal of assessing reproducibility in sufficiently sequenced samples (minimum number of footprints indicated on each respective plot).

To evaluate reproducibility in SOX2-FKBP mouse ES cells, CTCF-AID mouse ES cells and FOXA2-ΔHx hepatocytes, we calculated the Pearson’s correlation (with pearsonr from scipy) for effect sizes computed on a per-sample basis. This choice was intended to capture potential quantitative changes in cluster proportions between degron conditions and genotypes across replicates.

#### Identifying single-molecule co-occupancy patterns for footprint groups

Individual fibres with at least three Leiden-classified footprints within ±500 bp of repeat elements or TF motifs of interest were selected. Fibres with footprints greater than 200 nt and/or with footprints in the least abundant 10% of clusters were excluded from further analysis. Consecutive ‘triplet’ footprints were tabulated by computing midpoints and lengths for: (1) the central-most footprint nearest the repeat element or TF-binding motif; (2) the most proximal footprint upstream of the central-most footprint; and (3) the most proximal footprint downstream of the central-most footprint. Per-molecule accessibility data were computed for a 2-kb window centred on the repeat element or TF-binding motif of interest for visualization alongside footprint positions, accounting for sequence feature strand appropriately. Representative code for performing this analysis is provided (process_triplet_footprints.py).

#### Computing the enrichment of consecutive triplets on single chromatin fibres

Individual clusters were combined on the basis of groups defined by shared subfootprint organization from hierarchical clustering, as described above. Expected frequencies of footprint ‘triplets’ were computed as the product of the independent probabilities of observing each group within a given dataset. These values were used to calculate log_2_(observed (O)/expected (E)) ratios for each triplet across epigenomic domains, repeat elements and TF-binding motifs. Enrichment was assessed using Fisher’s exact test (fisher.test function in R), with false discovery rate (FDR) correction applied for multiple comparisons. The results were visualized as a ranked-order scatter plot, reflecting the effect size for each triplet.

#### Visualizing mean accessibility patterns at bound versus random TF motifs

Previously published scripts from our group were used to compute and cluster mean accessibility observed within ±750 bp of TF-binding motifs across different conditions (before and after TF knockdown; across genotypes)^3^. Euclidian distances for the resultant ORs for bound versus unbound motifs per sample are plotted to visualize their similarity across conditions and replicates.

#### Motif enrichment and classification

For each nucleosome, ±75 bp from the footprint centre was used as the input sequence for motif analyses. For each class of nucleosome, SEA^51^ was used to find enriched motifs within that nucleosome class, using the sequences from the total other nucleosomes as background. SEA was performed with default parameters using the JASPAR 2024 core non-redundant vertebrate motif database^85^. The resulting motifs were then assigned a class based on their JASPAR classification; classes with no representation across all nucleosome types were omitted. CentriMo analysis was performed using default parameters and significant and representative motifs were chosen to display^86^.

### Quantitative reproducibility and motivation for the architecture of the IDLI pipeline

Quantitative reproducibility information for all SAMOSA experiments and IDLI analyses presented in this paper is provided in Supplementary Figs. 1–8. Supplementary Fig. 1 visualizes the reproducibility of IDLI-based horizon plots from our various E14 SAMOSA replicate experiments, and quantitative estimates of reproducibility across replicates and within samples through bootstrapping. Supplementary Fig. 2 describes benchmarking analyses to ascertain the quality of CTCF-AID degron experiments, including visualizations of average single-molecule accessibility at CTCF sites, replicate reproducibility and examples of correlations between degradation and changes in the abundance of distinct single-molecule states, as previously described by our group^3,38,52^. Supplementary Fig. 3 is akin to Supplementary Fig. 2 but for SOX2-FKBP line experiments. Supplementary Fig. 4 is akin to Supplementary Fig. 2 but for FOXA2 perturbation experiments in hepatocytes. Supplementary Fig. 5 collates all dendrograms resulting from hierarchical clustering of nucleosome types to define groups. All dendrograms are labelled according to where nucleosome footprints were sampled from across the genome, and which sample was used for IDLI analysis. Supplementary Figs. 6 and 7 describe correlations between single-molecule accessibility patterns and nucleosome types, using CTCF (Supplementary Fig. 6) and SOX2 (Supplementary Fig. 7) as examples. This is provided as a reference to the reader to clarify that there are quantitative links between the overall accessibility state of the molecule (nucleosome-occluded motif versus open) and the abundance of distinct nucleosome types on those molecules. Supplementary Fig. 8 captures the quantitative reproducibility of all nucleosome-type definitions across each experiment presented in the manuscript.

We also include a Supplementary Note with the manuscript. This describes the usability of the IDLI approach and also motivates the parameter choices and computational design of the NN-HMM that underlies the IDLI pipeline.

Finally, all library metadata for sequencing experiments performed and analysed for this study are provided in Supplementary Table 1.

### Reporting summary

Further information on research design is available in the Nature Portfolio Reporting Summary linked to this article.

## Online content

Any methods, additional references, Nature Portfolio reporting summaries, source data, extended data, supplementary information, acknowledgements, peer review information; details of author contributions and competing interests; and statements of data and code availability are available at 10.1038/s41586-026-10418-6.

## Supplementary information

Supplementary Information Supplementary Figures 1–8, Supplementary Table 1 and Supplementary Note

Reporting Summary

## Data Availability

Raw and processed data are available at the Gene Expression Omnibus (GEO) under accession GSE288933. An example dataset for reproducing key findings is available via Zenodo at 10.5281/zenodo.18343575 (ref. ^87^).

## Code availability

Custom scripts used for analyses are available at https://github.com/RamaniLab/IDLI.
