# Supplementary material for: Pervasive and programmed nucleosome distortion on single chromatin fibres
Source: Nature. 2026 Apr 29;654(8118):513–22. doi: 10.1038/s41586-026-10418-6 (PMC13253354; doi:10.1038/s41586-026-10418-6)
Supplement: Supplementary file 1 — Supplementary Figures 1–8, Supplementary Table 1 and Supplementary Note [file 41586_2026_10418_MOESM1_ESM.pdf]

nature portfolio

https://doi.org/10.1038/s41586-026-10418-6

Supplementary information

Pervasive and programmed nucleosome distortion on single chromatin fibres

In the format provided by the authors and unedited

Supplementary Information Guide

Title: Pervasive and programmed nucleosome distortion on single chromatin fibers

Authors: Marty G Yang[1,16], Hannah J Richter[1,16], Simai Wang[1,16], Colin P McNally[1], Camille M Moore[1,2,3], Ali Emadi[4], Nicole E Harris[1], Simaron Dhillon[5,6], Michela Maresca[7,8], Huimin Pan[9,10], Hayden Saunders[2,3,11], Ruiqiao Yang[12,13], Megan S Ostrowski[1], Erika C Anderson[14], Elzo de Wit[7], Jacquelyn Maher[5,6], Yuhong Fan[12,13], Geeta J Narlikar[2], Elphege P Nora[2,14,15], Holger Willenbring[5,9,10], Hani Goodarzi[2,4#], Vijay Ramani[1,2,17#]

[1]: Gladstone Institute of Data Science and Biotechnology, Gladstone Institutes, San Francisco, CA 94158
[2]: Department of Biochemistry and Biophysics, University of California San Francisco, San Francisco, CA 94158
[3]: Tetrad Graduate Program, University of California, San Francisco, San Francisco, CA 94158
[4]: Arc Institute, Palo Alto, CA 94304
[5]: Liver Center, University of California, San Francisco, San Francisco, CA 94143
[6]: Division of Gastroenterology, Department of Medicine, University of California San Francisco, San Francisco, CA, 94143
[7]: Division of Gene Regulation, The Netherlands Cancer Institute, Amsterdam, Netherlands
[8]: Present Address: Department of Clinical Genetics, Erasmus MC University Medical Center, Rotterdam, Netherlands
[9]: Division of Transplant Surgery, Department of Surgery, University of California, San Francisco, San Francisco, CA, 94143
[10]: Eli and Edythe Broad Center of Regeneration Medicine and Stem Cell Research, University of California San Francisco, San Francisco, CA, 94143
[11]: Present Address: Department of Chemistry, University of California, Berkeley, Berkeley, CA 94720
[12]: School of Biological Sciences, Georgia Institute of Technology, Atlanta, GA 30332
[13]: Parker H. Petit Institute for Bioengineering and Bioscience, Georgia Institute of Technology, Atlanta, GA 30332
[14]: Cardiovascular Research Institute, UCSF, San Francisco, CA 94158
[15]: Chan-Zuckerberg BioHub, San Francisco, CA 94158
[16]: these authors contributed equally
[17]: lead contact
[#]: correspondence to hani.goodarzi@ucsf.edu & vijay.ramani@gladstone.ucsf.edu

Table of Contents

Section	Pages
Supplementary Figures 1-8…	2-10
Supplementary Table 1…	11-14
Supplementary Note….	15-17

Brief Description of Contents

Supplementary Figures 1-8: Quantitative reproducibility for all SAMOSA experiments and IDLI analyses presented in this paper.

Supplementary Table 1: Genomic coverage, read counts, and mono-di ratio for all Pacbio sequencing libraries used for analyses in this paper.

Supplementary Note: Notes on the usability of the IDLI approach, and parameter choices and computational design of the neural network – hidden Markov model (NN-HMM) underlying the IDLI pipeline.

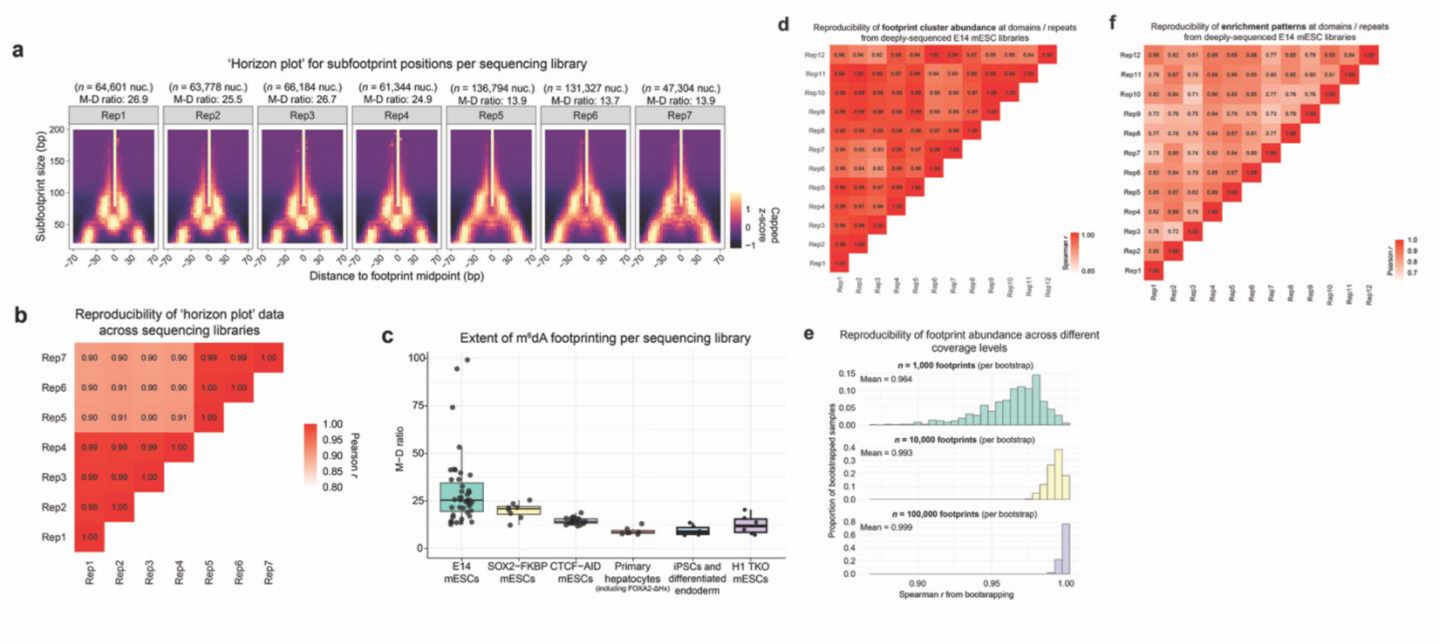

Supplementary Figure 1: Reproducibility and robustness of the IDLI pipeline across multiple footprinting experiments and samples presented in this study. A.) 'Horizon plots' derived from individual biological replicates, each with subtly different methylation extents, as indicated by the mononucleosome-dinucleosome ratio (MDR). The MDR is a quantitative measure of methylation extent in the SAMOSA footprinting assay. **B.)** Pearson *r* values from comparison of 'horizon plot' data across sequencing libraries. Samples with more similar MDRs exhibited quantitively higher levels of correlation in reproducibility of 'horizon plot' data (*e.g.* Rep1-4 vs. Rep5-7). **C.)** Box-and-whisker plots of MDR values for SAMOSA data included in this study, wherein individual points represent data from single biological replicates. Data are stratified based on cell type and genotype. Primary hepatocyte samples (wild-type and FOXA2-ΔHx) have slightly lower MDR values (MDR = 7.53 – 13.06), which we ascribe to slightly reduced efficiency of EcoGII footprinting in primary cells consequent to global differences in chromatin accessibility in pluripotent cells vs. terminally-differentiated cell types. **D.)** Spearman *r* values from comparison of proportions of nucleosome types at domains and repeats across biological replicates. **E.)** Bootstrapping analysis to determine number of footprints required for reproducible cluster assignments. From *n* = 795,765 domain- and repeat-associated footprints, we bootstrapped *n* = 1,000, 10,000, or 100,000 footprints per trial. Shown are the distribution of resultant Spearman *r* values (from a total of 10,000 trials per condition). These data suggest that our Leiden assignments for clusters of nucleosomal distortion patterns are reproducible and robust at the number of nucleosomes we have chosen for analysis. **F.)** Pearson *r* values from comparison of nucleosome type enrichment patterns across domains and repeats across biological replicates, related to **Figure 5A**.

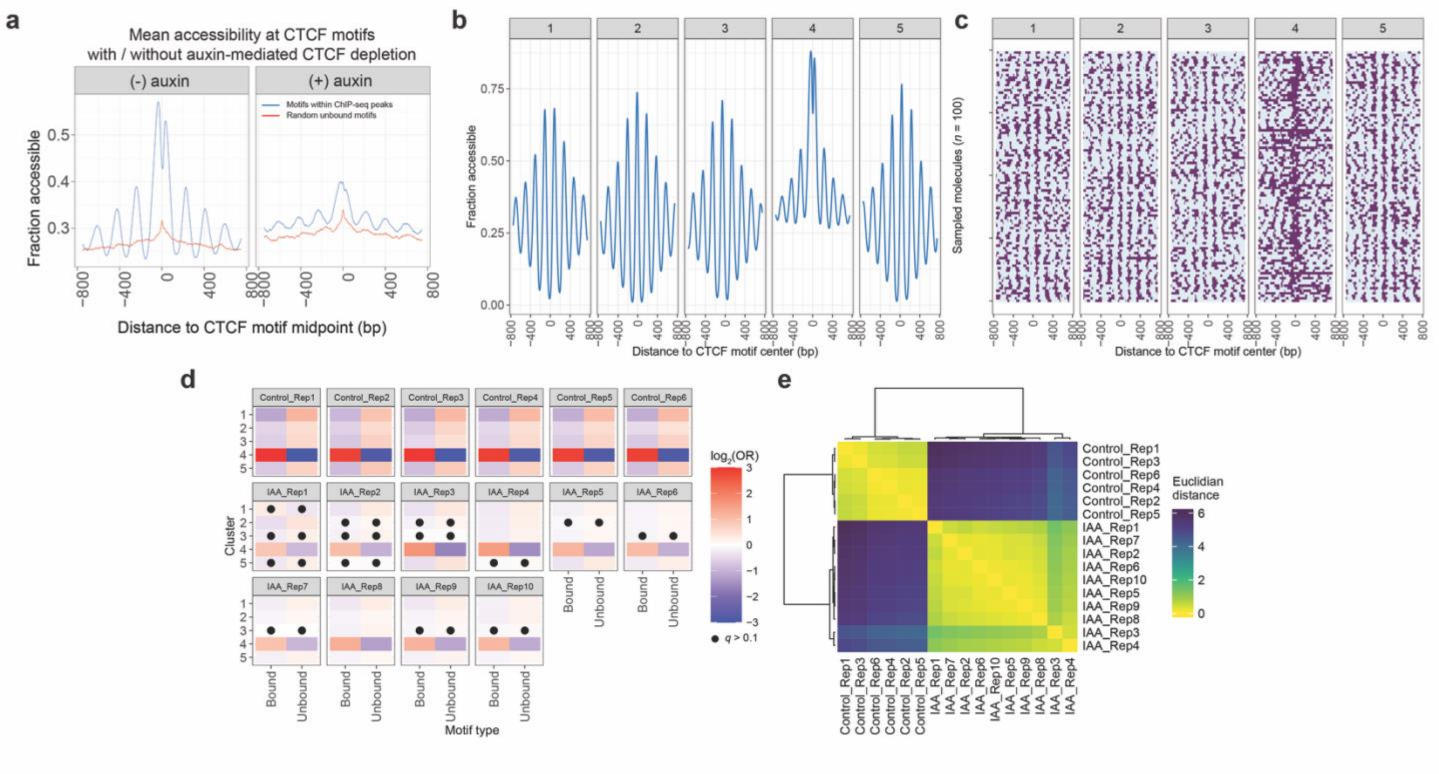

Supplementary Figure 2: Validation and reproducibility of CTCF-associated distortion patterns and data quality metrics. **A.)** Fraction accessible across ±750 bp window centered on bound (blue) and random (red) CTCF motifs. Comparison of data from auxin-treated and ddH$_2$O-treated CTCF-AID mESCs indicates that acute depletion of CTCF protein is associated with a loss in protection directly at bound CTCF motifs, a decrease in mean accessibility at bound CTCF loci, and an associated loss in phased nucleosomes flanking the nucleosome-free region. **B.)** Line plot representation of mean fraction accessible for Leiden-defined clusters at CTCF motifs in CTCF-AID mESCs. **C.)** Sampled single molecules underlying the averages shown as lineplots for each cluster. Each line represents 1,500 nucleotides extracted from an individual molecule, centered at the CTCF motif. **D.)** Heatmap representation of enrichment (red) or depletion (blue) of clusters comparing fibers at bound vs. unbound CTCF motifs. Data are shown for ddH$_2$O-treated and auxin-treated (designated as 'IAA) CTCF-AID mESC samples, stratified by biological replicate. Black dots mark Fisher's exact tests where $q > 0.1$ (not significant). **E.)** Heatmap of Euclidian distances computed from enrichment data in **(D)**; replicates cluster by drug treatment, suggesting that loss of CTCF protein is associated with a reproducible change in accessibility states at CTCF motif loci.

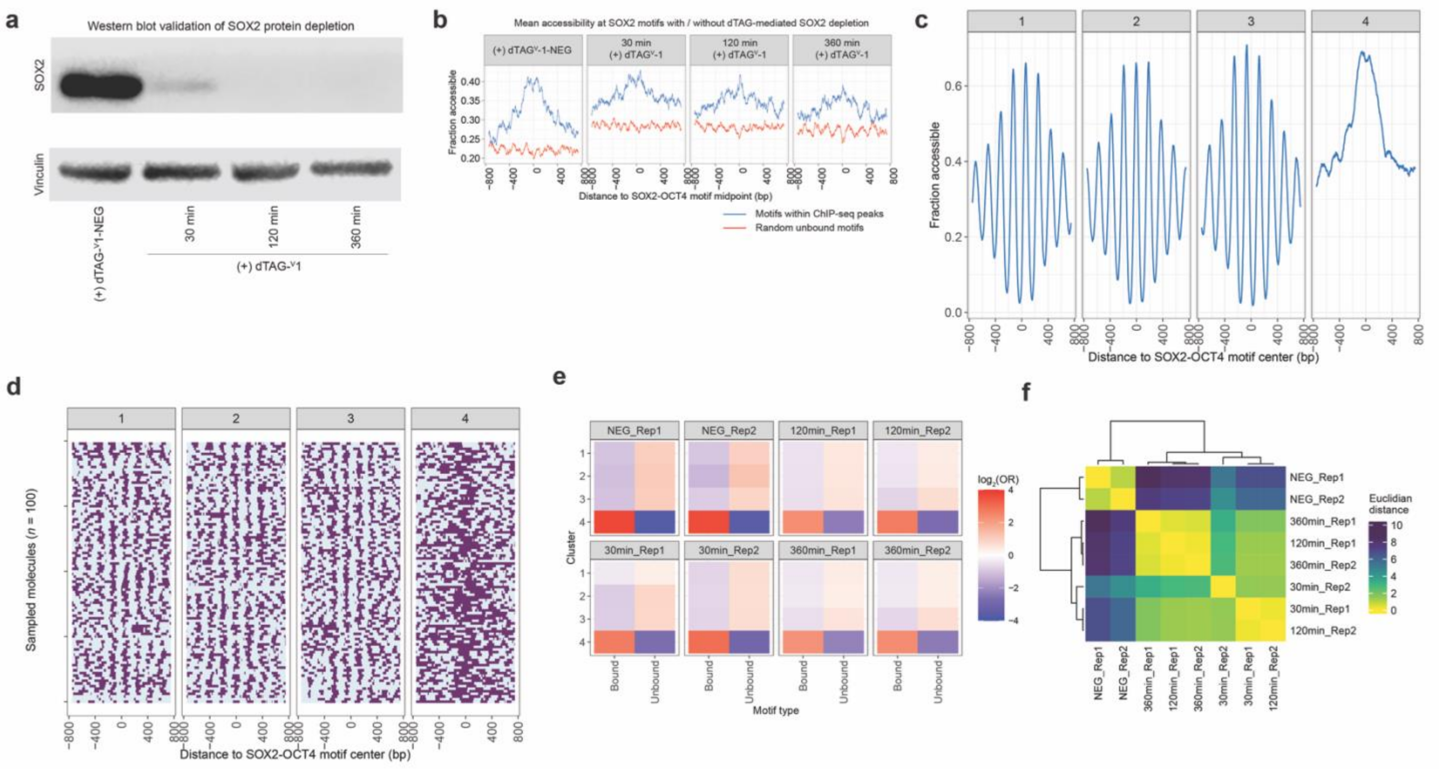

Supplementary Figure 3: Validation and reproducibility of SOX2-associated distortion patterns and data quality. A.) Western blot validation of dTAG-mediated degradation of SOX2 protein indicates near-complete degradation within 30 min of dTAG-^{V}1 treatment and undetectable levels of SOX2 by 2 hr and 6 hr. **B.)** Fraction accessible across ±750 bp window centered on bound (blue) and random (red) SOX2-OCT4 composite motifs. Comparison of data from dTAG^{V-}1-NEG- and dTAG^{V-}1-treated SOX2-FKBP mESCs indicates that acute depletion of SOX2 protein is associated with a slight decrease in chromatin accessibility at SOX2-OCT4 motif loci that fall within SOX2 ChIP-nexus peaks. **C.)** Line plot representation of mean fraction accessible for Leiden-defined clusters at SOX2-OCT4 composite motifs in SOX2-FKBP mESCs. **D.)** Sampled single molecules underlying the averages shown as lineplots for each cluster. Each line represents 1,500 nucleotides extracted from an individual molecule, centered at the SOX2-OCT4 motif. **E.)** Heatmap representation of enrichment (red) or depletion (blue) of clusters comparing fibers at bound vs. unbound SOX2-OCT4 composite motifs. Data are shown for dTAG^{V-}1-NEG- (designated as 'NEG) and dTAG^{V-}1-treated (time of treatment indicated) SOX2-FKBP mESC samples, stratified by biological replicate. Black dots mark Fisher's exact tests where $q > 0.1$ (not significant). **F.)** Heatmap of Euclidian distances computed from enrichment data in **(E)**; replicates cluster by drug treatment, suggesting that loss of SOX2 protein is associated with a reproducible change in accessibility states at SOX2-OCT4 motif loci.

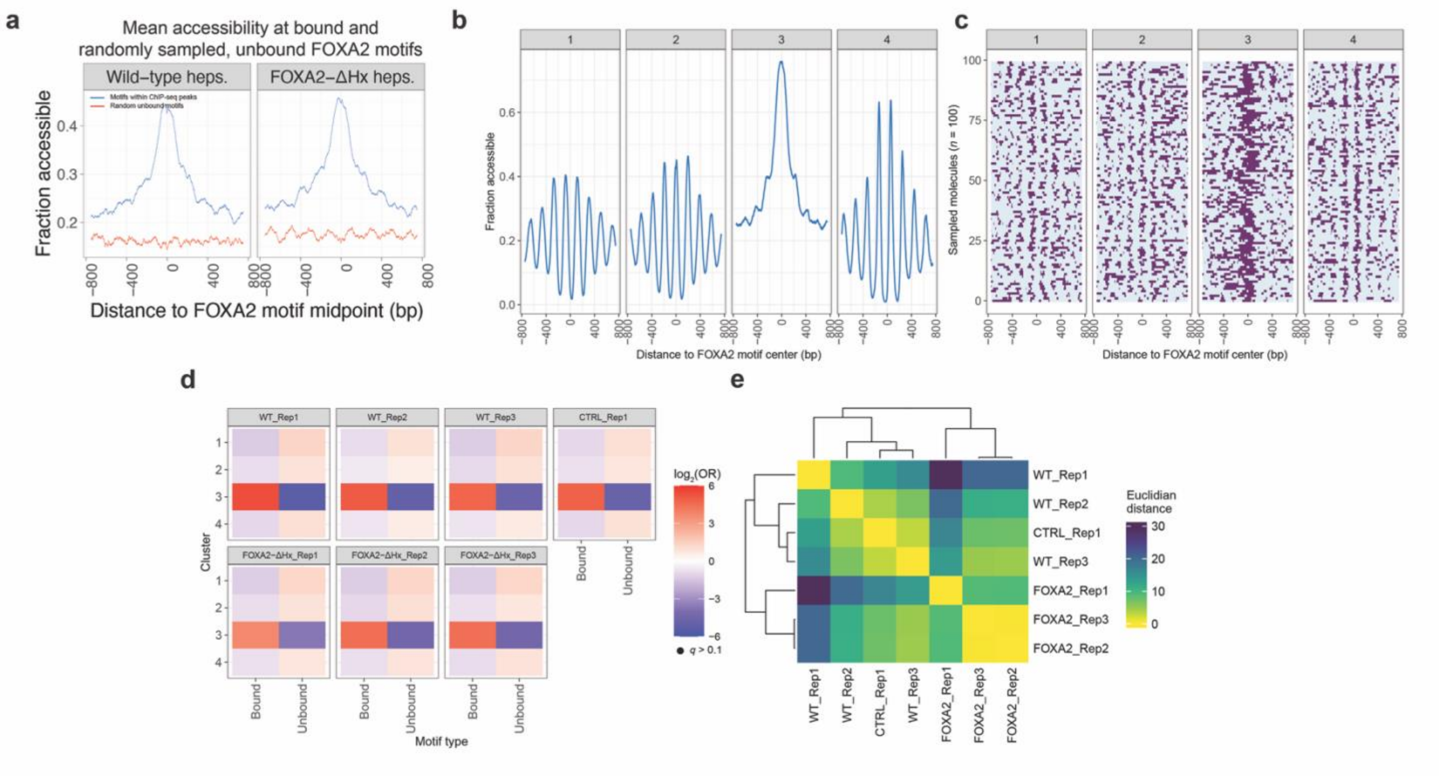

Supplementary Figure 4: Validation and reproducibility of FOXA2-associated distortion patterns and data quality metrics. **A.)** Fraction accessible across ±750 bp window centered on bound (blue) and random (red) FOXA2 motifs. Comparison of data from wild-type and heterozygous FOXA2-ΔHx hepatocytes indicates that deletion of a FOXA2 histone-interacting domain is associated with minimal changes in mean accessibility at cognate FOXA2 binding sites. **B.)** Line plot representation of mean fraction accessible for Leiden-defined clusters at FOXA2 motifs in wild-type and heterozygous FOXA2-ΔHx hepatocytes. **C.)** Sampled single molecules underlying the averages shown as lineplots for each cluster. Each line represents 1,500 nucleotides extracted from an individual molecule, centered at the FOXA2 motif. **D.)** Heatmap representation of enrichment (red) or depletion (blue) of clusters comparing fibers at bound vs. unbound FOXA2 motifs. Data are shown for wild-type (samples designated as WT are from C57BL/6J mice; sample designated as CTRL serves as littermate control for *Foxa2-ΔHx* samples) and heterozygous *Foxa2-ΔHx* hepatocyte samples, stratified by biological replicate. Black dots mark Fisher's exact tests where $q > 0.1$ (not significant). **E.)** Heatmap of Euclidian distances computed from enrichment data in **(D)**; replicates cluster by genotype, suggesting that accessibility patterns are more similar within wild-type samples than to helical-domain-mutant samples, and vice versa.

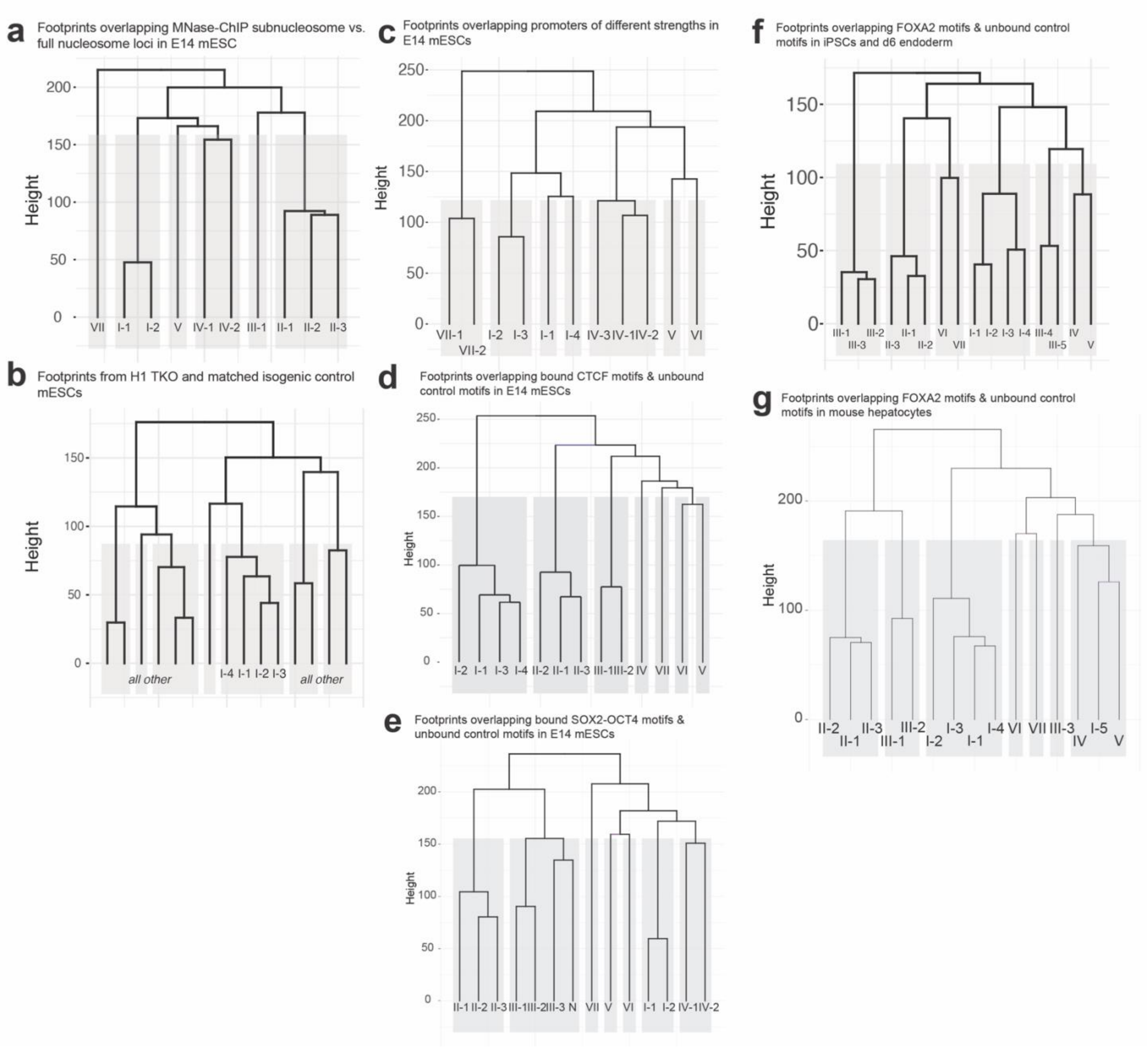

Supplementary Figure 5: Dendrograms for all nucleosome type clustering experiment, separated by factor-of-interest and cell type. A.) Dendrograms for nucleosome types resulting from hierarchical clustering of horizon plot patterns for footprints falling in MNase-ChIP defined regions enriched for subnucleosomes versus full nucleosomes. **B.)** Dendrograms for nucleosome types defined from footprinted H1 TKO and matched isogenic control mESCs. **C.)** Dendrograms for nucleosome types defined from footprints falling at mouse promoters, for the E14 mESC dataset. **D.)** Dendrograms for nucleosome types defined from footprints falling in the vicinity of ChIP-seq backed CTCF motifs and control CTCF motifs with no evidence of ChIP-seq signal (E14 mESC and CTCF-AID datasets). **E.)** Dendrograms for nucleosome types defined from footprints falling in the vicinity of SOX2-OCT4 composite motifs and unbound control motifs (E14 mESC and SOX2-FKBP datasets). **F.)** Dendrograms for nucleosome types defined from FOXA2 binding sites and control motifs (hiPSC and hEndoderm dataset). **G.)** Dendrograms for nucleosome types from FOXA2 binding sites and control motifs (mouse hepatocyte datasets [WT and *Foxa2*$^{+/\Delta Hx}$ mice]).

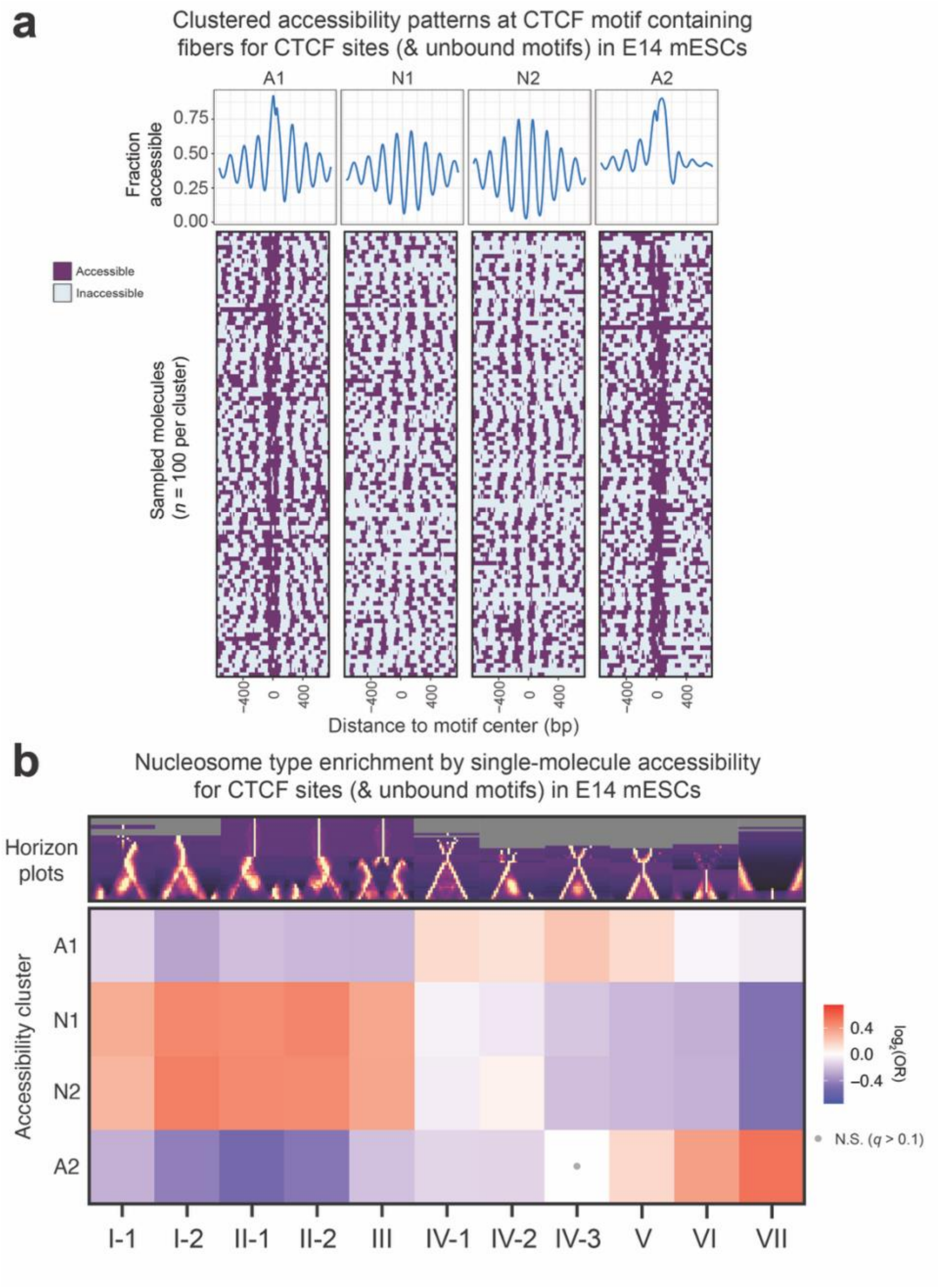

Supplementary Figure 6: Single-molecule chromatin accessibility patterns and nucleosome type calls at CTCF motifs in E14 mESC are correlated. A.) E14 mESC footprinted molecules falling over and centered at CTCF and control motifs, clustered by chromatin accessibility patterns, yielding 4 clusters (A1, N1, N2, and A2). A1 and A2 demonstrate focal accessibility / short footprints directly over the motif, while N1 and N2 demonstrate degrees of nucleosome occupancy over the motif. **B.)** Enrichment heatmap for nucleosome types defined for molecules falling within these clusters. N1 and N2 accessibility clusters are enriched for less accessible nucleosome types, while A1 and A2 molecules are enriched for more accessible nucleosome types, subnucleosomes, and very short methyltransferase protections.

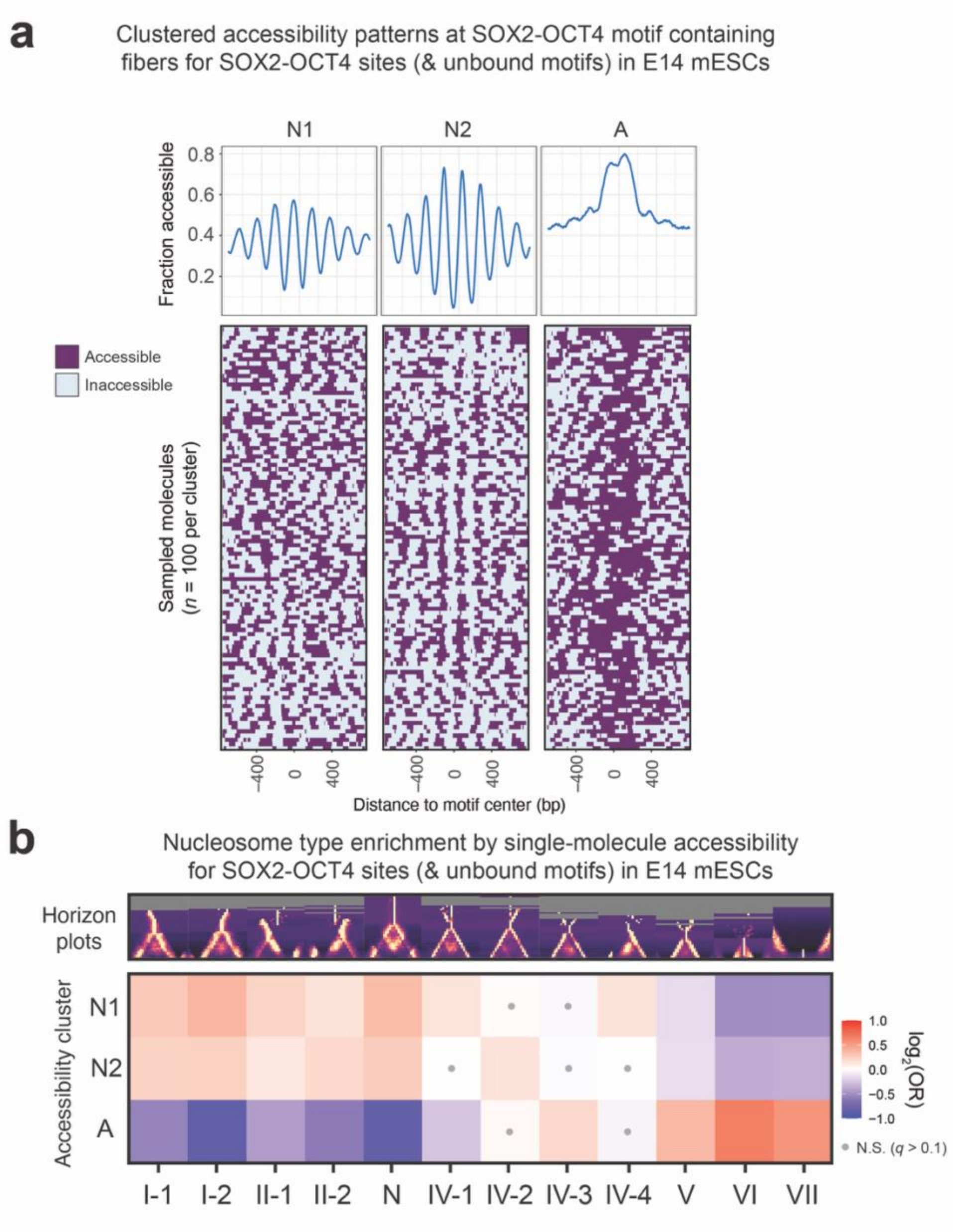

Supplementary Figure 7: Single-molecule chromatin accessibility patterns and nucleosome type calls at SOX2-OCT4 motifs in E14 mESC are correlated. A.) E14 mESC footprinted molecules falling over and centered at SOX2-OCT4 and control motifs, clustered by chromatin accessibility patterns, yielding 3 clusters (N1, N2, and A). A demonstrate focal accessibility directly over the motif, while N1 and N2 demonstrate degrees of nucleosome occupancy over the motif. **B.)** Enrichment heatmap for nucleosome types defined for molecules falling within these clusters. N1 and N2 accessibility clusters are enriched for less accessible nucleosome types, while A molecules are enriched for more accessible nucleosome types, subnucleosomes, and very short methyltransferase protections.

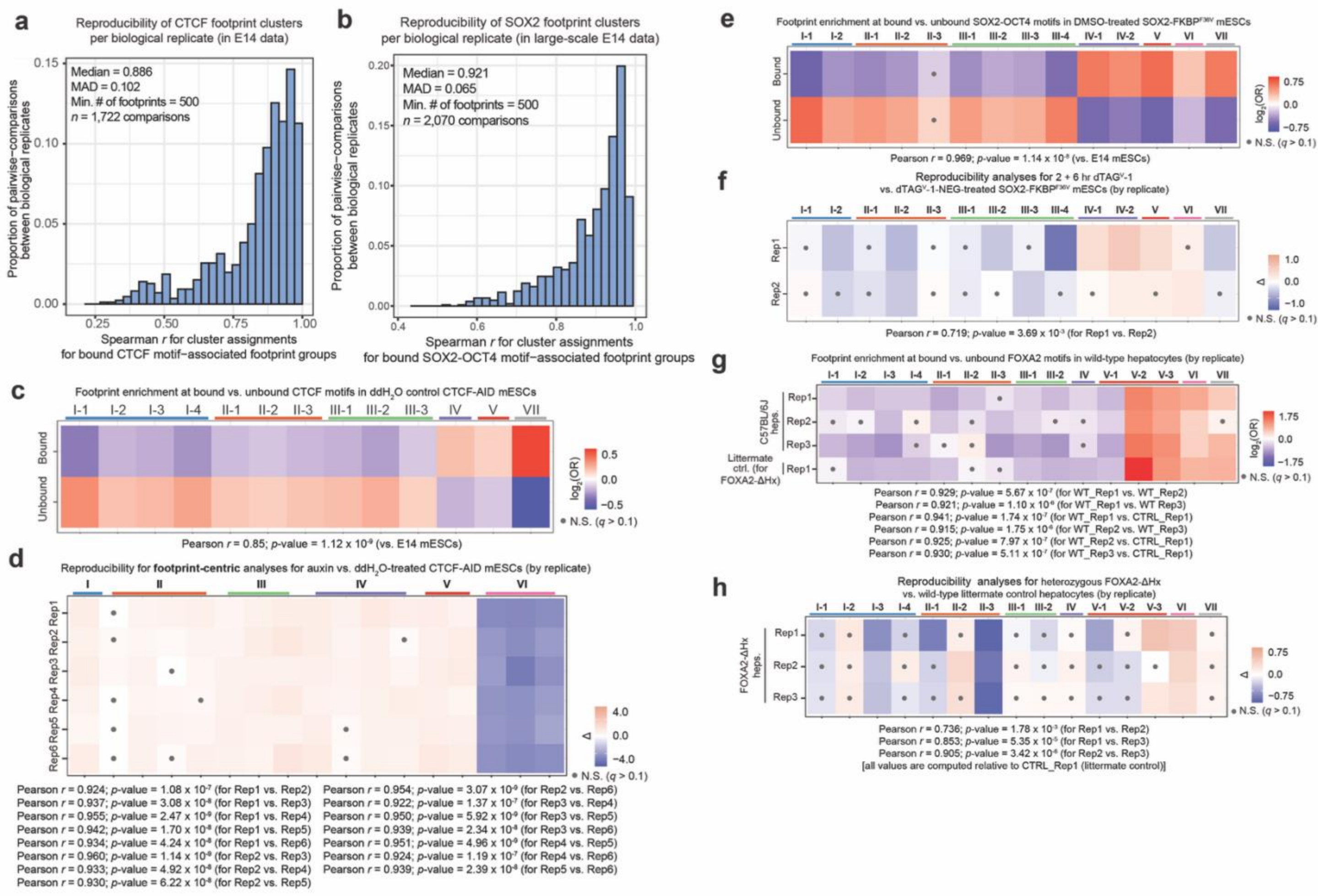

Supplementary Figure 8: Quantitative reproducibility of clustering data in E14 cells, between wildtype E14 cells and untreated degron lines, and for hepatocyte experiments. A.) Distribution of Spearman's *r* values for pairwise comparisons of cluster abundances for CTCF motif-associated distortion patterns in large-scale E14 mESC dataset. A minimum of *n* = 500 footprints was required in order to exclude lowly-sequenced libraries from this correlation analysis. **B.)** As in **(A)**, but for SOX2. **C.)** Heatmap of $\log_2$-transformed odds ratios (ORs) for assessing enrichment / depletion of different nucleosome types at bound vs. randomly sampled CTCF motifs in ddH_2O-treated CTCF-AID mESCs. ORs designated with a grey dot indicate those from Fisher's exact test that are not statistically significant (*i.e.* Storey *q*-value > 0.1). Correlation is computed relative to E14 mESC data (data from **Figure 3G**). **D.)** Heatmap of effect sizes (Δ) for nucleosome types at bound CTCF motifs in auxin vs. ddH_2O-treated CTCF-AID mESCs, stratified by biological replicate. Effect sizes designated with a grey dot indicate those from Fisher's exact test that are not statistically significant (*i.e.* Storey *q*-value > 0.1). **E.)** Heatmap of $\log_2$-transformed odds ratios (ORs) for assessing enrichment / depletion of different nucleosome types at bound vs. randomly sampled SOX2-OCT4 composite motifs in dTAG^{V}-1-NEG-treated SOX2-FKBP mESCs. ORs designated with a grey dot indicate those from Fisher's exact test that are not statistically significant (*i.e.* Storey *q*-value > 0.1). Correlation is computed relative to E14 mESC data. **F.)** Heatmap of effect sizes (Δ) for nucleosome types at bound SOX2-OCT4 motifs in dTAG^{V}-1-NEG vs. dTAG^{V}-1-treated (for 2 and 6 hr) SOX2-FKBP mESCs, stratified by biological replicate. Effect sizes designated with a grey dot indicate those from Fisher's exact test that are not statistically significant (*i.e.* Storey *q*-value > 0.1). **G.)** Heatmap of $\log_2$-transformed odds ratios (ORs) for assessing enrichment / depletion of different nucleosome types at bound vs. randomly sampled FOXA2 motifs in wild-type hepatocytes, stratified by biological replicate. ORs designated with a grey dot indicate those from Fisher's exact test that are not statistically significant (*i.e.* Storey *q*-value > 0.1). **H.)** Heatmap of effect sizes (Δ) for nucleosome types at

bound FOXA2 motifs in heterozygous FOXA2-ΔHx hepatocytes vs. littermate control, stratified by biological replicate. Effect sizes designated with a grey dot indicate those from Fisher's exact test that are not statistically significant (*i.e.* Storey *q*-value > 0.1).

Supplementary Table 1

GEO_NAME	COVERAGE_Gb	READ_COUNT	MD_RATIO
E14_mESC_Rep1_Seq1	1.7397	392507	14.2086
E14_mESC_Rep2_Seq1	1.5321	364144	13.6378
E14_mESC_Rep3_Seq1	0.3185	211256	26.9156
E14_mESC_Rep4_Seq1	0.2887	191028	25.5006
E14_mESC_Rep5_Seq1	0.2689	174345	26.6746
E14_mESC_Rep6_Seq1	0.2316	152185	24.8978
E14_mESC_Rep7_Seq1	1.0006	507515	13.9439
E14_mESC_Rep8_Seq1	0.8386	417810	13.7238
E14_mESC_Rep9_Seq1	1.5908	767729	13.8820
E14_mESC_Rep10_Seq1	0.0480	102854	15.4678
E14_mESC_Rep11_Seq1	0.0369	77411	29.4766
E14_mESC_Rep12_Seq1	0.0459	115446	25.4437
E14_mESC_Rep13_Seq1	0.0451	108519	32.7797
E14_mESC_Rep14_Seq1	0.0364	65953	28.4595
E14_mESC_Rep10_Seq2	0.0436	97253	15.4678
E14_mESC_Rep11_Seq2	0.0302	66197	29.4766
E14_mESC_Rep12_Seq2	0.0177	43220	25.4437
E14_mESC_Rep13_Seq2	0.0203	47612	32.7797
E14_mESC_Rep14_Seq2	0.0429	87113	28.4595
E14_mESC_Rep15_Seq1	1.7151	458212	17.0346
E14_mESC_Rep16_Seq1	1.0138	294345	12.2757
E14_mESC_Rep17_Seq1	1.2248	335034	21.2119
E14_mESC_Rep18_Seq1	1.2191	323599	22.5091
E14_mESC_Rep19_Seq1	0.1563	19134	19.0015
E14_mESC_Rep20_Seq1	0.1482	18217	21.6413
E14_mESC_Rep21_Seq1	0.4448	96279	17.5838
E14_mESC_Rep22_Seq1	0.5942	131037	19.9605
E14_mESC_Rep23_Seq1	0.2220	39032	25.7841
E14_mESC_Rep24_Seq1	0.1729	63155	16.4741
E14_mESC_Rep25_Seq1	0.1594	27066	24.6330
E14_mESC_Rep26_Seq1	0.1419	51294	12.7255
E14_mESC_Rep27_Seq1	0.2098	39340	24.5392
E14_mESC_Rep28_Seq1	0.2069	68591	23.1288
E14_mESC_Rep29_Seq1	0.2157	37082	24.7726
E14_mESC_Rep23_Seq2	1.6146	281851	26.0405
E14_mESC_Rep24_Seq2	1.2885	342608	17.6149
E14_mESC_Rep25_Seq2	0.8645	150668	25.3845
E14_mESC_Rep26_Seq2	0.9552	277085	12.8929
E14_mESC_Rep28_Seq2	1.3176	359677	22.5742
E14_mESC_Rep29_Seq2	1.4633	250173	23.2177

E14_mESC_Rep30_Seq1	1.4063	228556	41.3475
E14_mESC_Rep31_Seq1	1.3885	235494	36.3402
E14_mESC_Rep32_Seq1	1.1791	202616	21.5882
E14_mESC_Rep33_Seq1	1.3571	231895	41.3151
E14_mESC_Rep34_Seq1	1.2798	204961	36.1563
E14_mESC_Rep35_Seq1	1.1437	193456	53.3068
E14_mESC_Rep36_Seq1	1.5765	257962	38.6260
E14_mESC_Rep37_Seq1	1.1393	189602	29.9794
E14_mESC_Rep38_Seq1	1.1185	183971	25.4016
E14_mESC_Rep39_Seq1	1.3715	244349	39.7776
E14_mESC_Rep40_Seq1	1.2023	206373	30.3785
E14_mESC_Rep41_Seq1	1.4225	240222	41.6463
E14_mESC_Rep30_Seq2	1.4417	227336	41.2174
E14_mESC_Rep31_Seq2	1.4007	231073	36.2297
E14_mESC_Rep32_Seq2	1.2156	202772	21.2369
E14_mESC_Rep33_Seq2	1.3840	229912	40.6531
E14_mESC_Rep34_Seq2	1.3275	206371	36.0386
E14_mESC_Rep35_Seq2	1.1534	189825	55.1256
E14_mESC_Rep36_Seq2	1.6154	256550	39.5210
E14_mESC_Rep37_Seq2	1.1573	187181	30.3166
E14_mESC_Rep38_Seq2	1.1443	182562	25.4275
E14_mESC_Rep39_Seq2	1.3577	235799	39.2618
E14_mESC_Rep40_Seq2	1.2221	203862	29.9690
E14_mESC_Rep41_Seq2	1.4580	238905	41.1673
E14_mESC_Rep30_Seq3	1.4660	227566	40.5161
E14_mESC_Rep31_Seq3	1.4122	229776	35.6302
E14_mESC_Rep32_Seq3	1.2272	201878	21.1592
E14_mESC_Rep33_Seq3	1.3991	229372	40.4388
E14_mESC_Rep34_Seq3	1.3497	206793	35.1610
E14_mESC_Rep35_Seq3	1.1650	189239	53.4912
E14_mESC_Rep36_Seq3	1.6459	257411	39.0073
E14_mESC_Rep37_Seq3	1.1795	188078	30.5935
E14_mESC_Rep38_Seq3	1.1622	182490	25.3669
E14_mESC_Rep39_Seq3	1.3590	233104	39.3427
E14_mESC_Rep40_Seq3	1.2293	202734	29.5927
E14_mESC_Rep41_Seq3	1.4817	239479	41.3953
E14_mESC_Rep30_Seq4	1.4960	237105	40.1796
E14_mESC_Rep31_Seq4	1.4592	241944	35.5017
E14_mESC_Rep32_Seq4	1.2802	214200	20.8878
E14_mESC_Rep33_Seq4	1.4431	240812	40.0839
E14_mESC_Rep34_Seq4	1.3770	214847	35.7561
E14_mESC_Rep35_Seq4	1.2015	198922	55.4150

E14_mESC_Rep36_Seq4	1.6846	268727	38.8353
E14_mESC_Rep37_Seq4	1.2136	197033	30.3262
E14_mESC_Rep38_Seq4	1.1983	191815	25.2469
E14_mESC_Rep39_Seq4	1.4228	248160	39.4919
E14_mESC_Rep40_Seq4	1.2794	214568	29.8565
E14_mESC_Rep41_Seq4	1.5105	249101	42.2140
E14_mESC_Rep42_Seq1	0.4163	95832	10.2642
E14_mESC_Rep43_Seq1	0.2666	90478	99.1250
E14_mESC_Rep44_Seq1	0.0788	32850	94.4658
E14_mESC_Rep45_Seq1	0.4740	123131	74.1681
CTCF_mESC_auxin_Rep7_Seq1	0.1568	22372	15.2692
CTCF_mESC_auxin_Rep8_Seq1	0.1833	25777	14.9669
CTCF_mESC_auxin_Rep9_Seq1	0.4853	127072	13.7510
CTCF_mESC_auxin_Rep10_Seq1	0.5477	134270	13.4124
CTCF_mESC_DMSO_Rep1_Seq1	0.1228	27750	13.3316
CTCF_mESC_DMSO_Rep2_Seq1	0.1162	27187	13.9438
CTCF_mESC_DMSO_Rep3_Seq1	0.1223	29110	13.4493
CTCF_mESC_DMSO_Rep4_Seq1	0.0877	20444	13.1629
CTCF_mESC_DMSO_Rep5_Seq1	0.1181	25832	12.3893
CTCF_mESC_DMSO_Rep6_Seq1	0.1467	34190	11.8338
CTCF_mESC_DMSO_Rep1_Seq2	1.4429	234867	13.7507
CTCF_mESC_DMSO_Rep2_Seq2	1.4153	232445	14.3495
CTCF_mESC_DMSO_Rep3_Seq2	1.3036	219137	14.7703
CTCF_mESC_DMSO_Rep4_Seq2	1.5518	250734	14.1364
CTCF_mESC_DMSO_Rep5_Seq2	1.4017	222853	13.2624
CTCF_mESC_DMSO_Rep6_Seq2	1.6134	268549	12.3981
CTCF_mESC_auxin_Rep1_Seq1	1.2944	195319	14.2573
CTCF_mESC_auxin_Rep2_Seq1	1.3337	207372	16.7407
CTCF_mESC_auxin_Rep3_Seq1	1.2564	173906	15.8862
CTCF_mESC_auxin_Rep4_Seq1	1.5479	228009	15.9054
CTCF_mESC_auxin_Rep5_Seq1	1.4449	194591	16.1056
CTCF_mESC_auxin_Rep6_Seq1	1.2935	181375	15.7330
CTCF_mESC_auxin_Rep1_Seq2	1.4429	234867	19.4458
CTCF_mESC_auxin_Rep2_Seq2	1.4153	232445	18.1645
CTCF_mESC_auxin_Rep3_Seq2	1.3036	219137	16.5215
CTCF_mESC_auxin_Rep4_Seq2	1.5518	250734	16.7412
CTCF_mESC_auxin_Rep5_Seq2	1.4017	222853	16.8573
CTCF_mESC_auxin_Rep6_Seq2	1.6134	268549	15.6948
SOX2_mESC_dTAG_2hr_Rep1_Seq1	1.8378	195662	23.5683
SOX2_mESC_dTAG_2hr_Rep2_Seq1	1.7879	197937	20.4780
SOX2_mESC_dTAG_30min_Rep1_Seq1	1.7822	193602	25.4684
SOX2_mESC_dTAG_30min_Rep2_Seq1	1.9508	218289	21.4642

SOX2_mESC_dTAG_6hr_Rep1_Seq1	1.5417	164816	18.5211
SOX2_mESC_dTAG_6hr_Rep2_Seq1	1.4469	158410	21.5698
SOX2_mESC_DMSO_Rep1_Seq1	1.5368	173356	12.3228
SOX2_mESC_DMSO_Rep2_Seq1	1.6977	187384	16.3177
C57BL6J_mHep_Rep1_Seq1	1.4709	252151	8.3183
C57BL6J_mHep_Rep3_Seq1	1.7967	269214	8.6765
C57BL6J_mHep_Rep2_Seq1	0.5404	149786	13.0587
Foxa2_dHx_mHep_Rep1_Seq1	2.9903	323307	10.3382
Foxa2_dHx_mHep_Rep3_Seq1	1.7344	193265	8.2771
Foxa2_control_mHep_Rep1_Seq1	1.2586	186146	7.5359
Foxa2_dHx_mHep_Rep2_Seq1	1.4345	181607	7.7929
NIPBL_mESC_dTAG_Rep1_Seq1	0.0775	11126	19.0845
NIPBL_mESC_dTAG_Rep2_Seq1	0.0048	768	18.0476
NIPBL_mESC_dTAG_Rep3_Seq1	0.0975	16178	21.6214
NIPBL_mESC_dTAG_Rep4_Seq1	0.0639	10179	17.4567
NIPBL_mESC_DMSO_Rep1_Seq1	0.0603	10108	18.2944
NIPBL_mESC_DMSO_Rep2_Seq1	0.0094	1444	17.1053
NIPBL_mESC_dTAG_Rep1_Seq2	2.2901	300579	23.5281
NIPBL_mESC_dTAG_Rep2_Seq2	2.0897	316815	18.3811
NIPBL_mESC_dTAG_Rep3_Seq2	2.4031	357648	20.7054
NIPBL_mESC_dTAG_Rep4_Seq2	2.3041	310108	19.2577
NIPBL_mESC_DMSO_Rep1_Seq2	2.2583	317316	18.8228
NIPBL_mESC_DMSO_Rep2_Seq2	1.6676	264802	20.1321
ENDO_D0_Rep1_Seq1	0.8211	84738	8.3958
ENDO_D0_Rep2_Seq1	1.1205	112466	7.2743
ENDO_D0_Rep3_Seq1	1.0141	94896	7.0233
ENDO_D6_Rep1_Seq1	0.8567	85356	11.6352
ENDO_D6_Rep2_Seq1	0.9782	102620	9.2151
ENDO_D6_Rep3_Seq1	1.1118	91876	13.5749
H1_WT_Rep1_Seq1	1.2871	284416	9.9462
H1_WT_Rep2_Seq1	1.1345	283092	7.2156
H1_WT_Rep3_Seq1	0.9123	217451	8.0385
H1_TKO_Rep1_Seq1	1.9286	438228	20.4098
H1_TKO_Rep2_Seq1	0.9472	304582	13.8191
H1_TKO_Rep3_Seq1	0.9435	263397	16.0469

Supplementary Table 1: Quantification of key metrics for sequencing data used. Column “GEO_NAME” is a brief description of the data, matching the name used in the GEO deposition. Column “COVERAGE_Gb” reports the size of the sequencing library in 10^9 aligned bases. Column “READ_COUNT” reports the number of aligned sequencing reads for each library. Column “MD_RATIO” reports the mono-di ratio for SAMOSA-based nucleosome footprinting, a measure of methylation extent.

Supplementary Note

(1) Implementing SAMOSA and the IDLI nucleosome type classification pipeline

Our approach can be adopted by any lab with access to the NEB catalog (we use the commercially available enzyme EcoGII), GitHub, and a PacBio sequencer (either Revio or Sequel II). In terms of computational resources, inference can be run on standard CPU clusters, while model training (which only needs to be performed once to learn the specific biases associated with each different enzyme being used) requires GPU access to train models quickly. Even then, compared to more recent NN models, those used in our publications for m^6dA classification are lightweight.

(2) Motivating the IDLI architecture

IDLI uses a 'hybrid' neural network – hidden markov model (NN-HMM) architecture to define subnucleosome-resolution protein-DNA interactions. Our deliberate use of variable transition probability *t* is a principled modeling decision, not a heuristic tuning of hidden layers. In our framework, it controls the expected dwell time in each HMM state— accessible or inaccessible, and allows us to detect structural features at different physical length scales. At lower *t* values (*e.g.* *t* = 0.001), the model favors longer contiguous inaccessible regions consistent with fully wrapped nucleosomes, while higher *t* values (*e.g.* *t* = 0.01) enable detection of shorter protected regions consistent with subnucleosomal particles. This approach is conceptually analogous to multi-scale decomposition in signal processing, and allows us to recover a richer and more nuanced view of chromatin structure without redefining the underlying states.

Importantly, the underlying hidden states remain binary (accessible or inaccessible) and biologically interpretable across all *t*, with *t* acting as a scale selector (*i.e.* a 'tuning knob') for resolution at which the model operates, rather than a latent-layer exposure that may cause noise/bias. To demonstrate the value of our NN-HMM implementation compared to a more traditional approach, we ran the forward-backward algorithm on our data to compute posterior state probabilities. These posterior values give us a quantitative measure of uncertainty for each base position. As shown in **Figure S1A-C**, for three random molecules, the model consistently returns high-confidence predictions, mostly either 0 (inaccessible) or 1 (accessible). This is true even at higher *t*, supporting the robustness of our state calls across different resolutions.

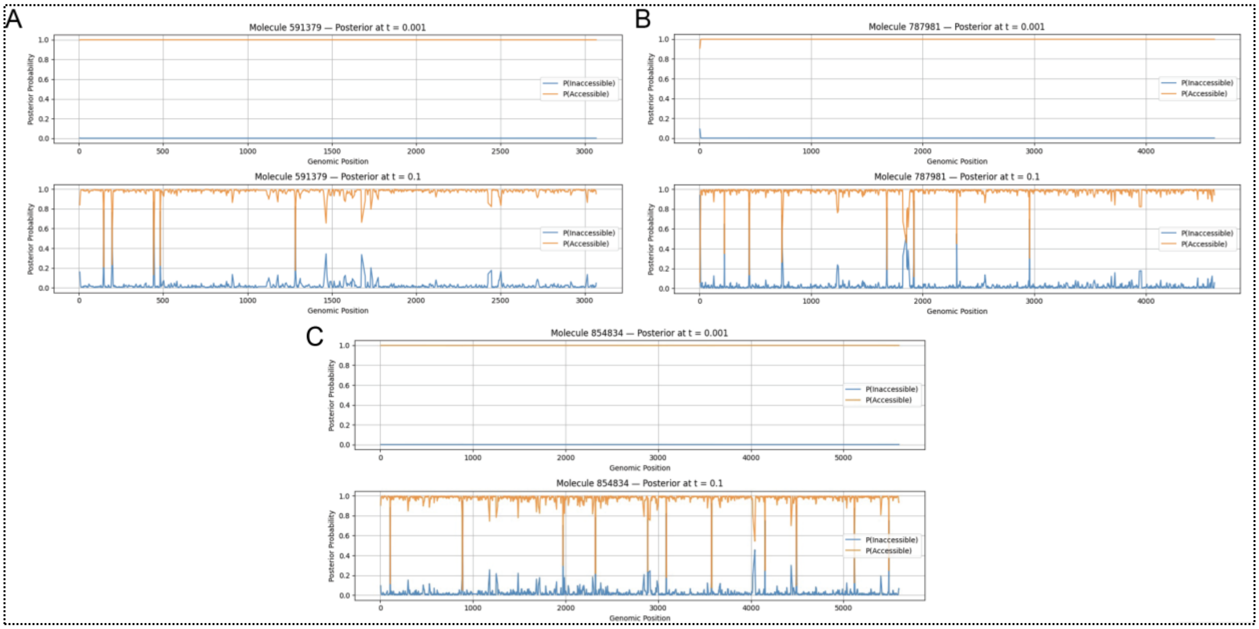

Figure S1: Posterior probabilities calculated at multiple *t* values for three randomly sampled, footprinted molecules. (A-C) Posterior probabilities calculated at *t* = 0.001 and *t* = 0.1 for three randomly sampled molecules from E14 mESCs. Even when *t* spans a large range of values, resulting models return high-confidence predictions either close to 1 or close to 0.

(3) On selecting an appropriate range of* t *values

Our NN-HMM architecture is also "non-traditional" in that the transition probability *t* is manually defined by the user, as opposed to being 'learned' via expectation-maximization (EM). This is in part because the emission probabilities of the HMM are pre-defined through a neural network, but we nevertheless sought to motivate that the *t* range of values used in our manuscript are defensible. To do so, we took advantage of a data-driven approach to validate our choice of *t* range. Specifically, we used a Baum-Welch EM for to learn state transition probabilities for a single sampled molecule. These "effective ***t"*** values were then derived from the learned matrices of inferred *t*, $(1 - \underline{P}(stay))$, where $\underline{P}(stay)$ is the average probability of stay.

In details, let $\boldsymbol{A} \in \boldsymbol{R}^{2\times 2}$ be the transition matrix of state learned by Baum-Welch (EM):

$$A = \begin{bmatrix} P_{00} & P_{01} \\ P_{10} & P_{11} \end{bmatrix}$$

P_{00} = Probability of staying in **inaccessible** state

P_{11} = Probability of staying in **accessible** state

$P_{01} = 1 - P_{00}$, Probability of transition from **inaccessible** to **accessible** state

$P_{10} = 1 - P_{11}$, Probability of transition from **accessible** to **inaccessible** state

And then, to be able to derive an effective transition probability ***t*** as the average probability of switching states, equivalent to the fixed-***t*** used in our HMM framework, we define ***Inferred t***, as below:

$$\boldsymbol{t} = 1 - \bar{P}(stay) = 1 - \frac{P_{00} + P_{11}}{2}$$

For instance, below is the definition and derivation for one molecule (molecule 191):

$$A = \begin{bmatrix} 0.9679 & 0.0321 \\ 0.0045 & 0.9955 \end{bmatrix} \quad \Rightarrow \quad \boldsymbol{t} = 1 - \frac{0.9679 + 0.9955}{2} = 0.0183$$

where this value lies well within our fixed-*t* we explored (*t* = 0.001 - 0.1010). This approach thus validates that our chosen *t* values are reasonable representative transition probabilities.

We sought to further validate this analysis by studying more randomly sampled molecules. We performed EM on 20 additional randomly picked molecules, and plotted the distribution of inferred *t* values (**Figure S2**). The inferred-*t* values varied across a broad range, reflecting the biological heterogeneity of chromatin accessibility. We visualized the distribution of these EM-inferred *t* across these 20 molecules overlaid with a Kernel Density Estimate (KDE)-smoothed curve (Blue curve in the figure), ranging from ~0.05 to ~0.36, and centered at ~ 0.12.

Excitingly, the majority of these values fall in the range of *t* values we used in our HMM analysis (*t* = 0.001 - 0.1010), supporting the biological relevance and robustness of our framework.

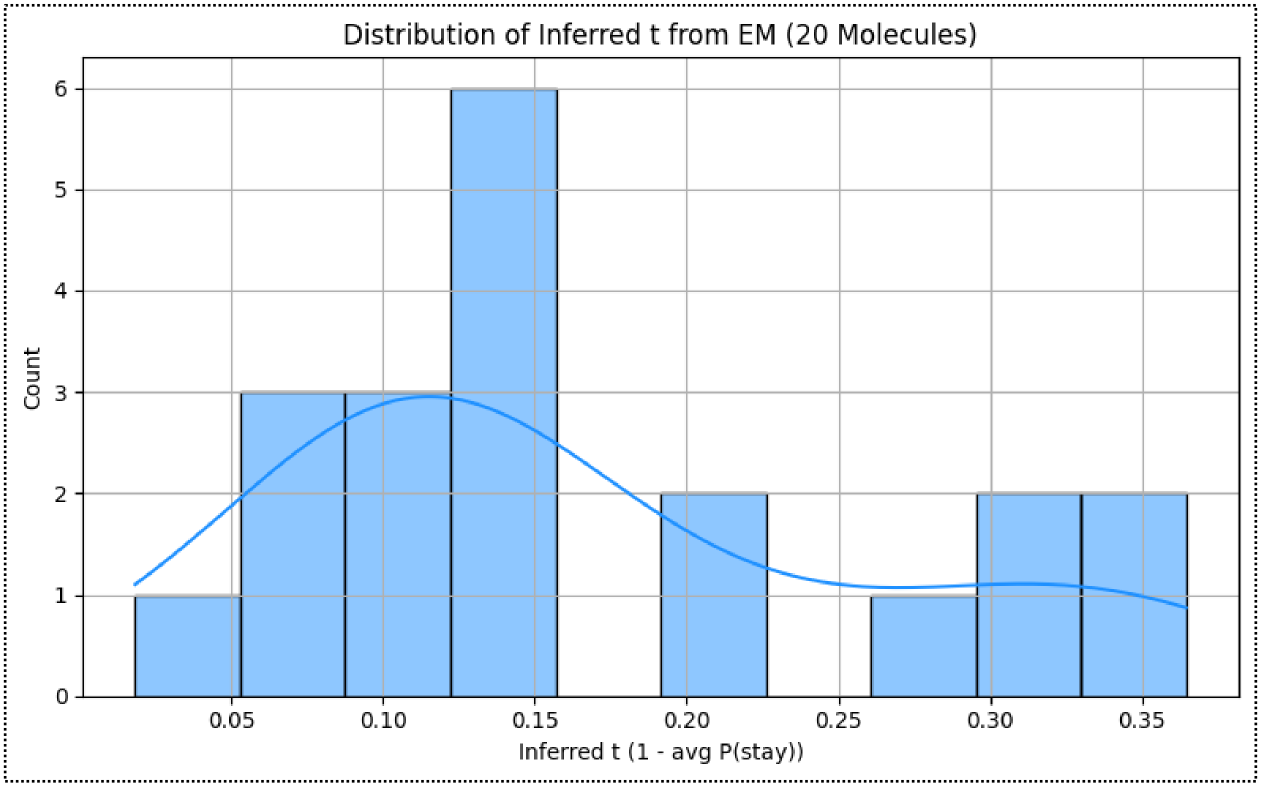

Figure S2: Distribution of inferred *t* from Baum-Welch EM for 20 sampled SAMOSA molecules. As described above, we ran Baum-Welch inference across 20 sampled, footprinted fibers, and plotted the resulting inferred *t* values, which represent 1 – average(P(stay)).
