# Supplementary material for: Pervasive and programmed nucleosome distortion on single chromatin fibres
Source: Nature. 2026 Apr 29;654(8118):513–22. doi: 10.1038/s41586-026-10418-6 (PMC13253354; doi:10.1038/s41586-026-10418-6)
Supplement: Supplementary file 2 — Reporting Summary [file 41586_2026_10418_MOESM2_ESM.pdf]

nature portfolio

Corresponding author(s): Hani Goodarzi & Vijay Ramani

Last updated by author(s): Dec 6, 2026

Reporting Summary

Nature Portfolio wishes to improve the reproducibility of the work that we publish. This form provides structure for consistency and transparency in reporting. For further information on Nature Portfolio policies, see our Editorial Policies and the Editorial Policy Checklist.

Statistics

For all statistical analyses, confirm that the following items are present in the figure legend, table legend, main text, or Methods section.

n/a	Confirmed	
☐	☒	The exact sample size (*n*) for each experimental group/condition, given as a discrete number and unit of measurement
☐	☒	A statement on whether measurements were taken from distinct samples or whether the same sample was measured repeatedly
☐	☒	The statistical test(s) used AND whether they are one- or two-sided<br>*Only common tests should be described solely by name; describe more complex techniques in the Methods section.*
☒	☐	A description of all covariates tested
☒	☐	A description of any assumptions or corrections, such as tests of normality and adjustment for multiple comparisons
☐	☒	A full description of the statistical parameters including central tendency (e.g. means) or other basic estimates (e.g. regression coefficient) AND variation (e.g. standard deviation) or associated estimates of uncertainty (e.g. confidence intervals)
☐	☒	For null hypothesis testing, the test statistic (e.g. *F*, *t*, *r*) with confidence intervals, effect sizes, degrees of freedom and *P* value noted<br>*Give P values as exact values whenever suitable.*
☒	☐	For Bayesian analysis, information on the choice of priors and Markov chain Monte Carlo settings
☒	☐	For hierarchical and complex designs, identification of the appropriate level for tests and full reporting of outcomes
☐	☒	Estimates of effect sizes (e.g. Cohen's *d*, Pearson's *r*), indicating how they were calculated

Our web collection on statistics for biologists contains articles on many of the points above.

Software and code

Policy information about availability of computer code

Data collection	Sequencing data was collected using a Pacific Biosciences Sequel II running SMRTlink 11.1.0.166339. Raw data was processed using ccs (Pacific Biosciences, v6.4.0) and demultiplexed using lima (Pacific Biosciences, v2.1.0).
Data analysis	Refer to https://github.com/RamaniLab/IDLI for details on data analysis.

For manuscripts utilizing custom algorithms or software that are central to the research but not yet described in published literature, software must be made available to editors and reviewers. We strongly encourage code deposition in a community repository (e.g. GitHub). See the Nature Portfolio guidelines for submitting code & software for further information.

Data

Policy information about availability of data

All manuscripts must include a data availability statement. This statement should provide the following information, where applicable:

- Accession codes, unique identifiers, or web links for publicly available datasets
- A description of any restrictions on data availability
- For clinical datasets or third party data, please ensure that the statement adheres to our policy

Raw data and processed data is at GEO accession GSE288933.

Research involving human participants, their data, or biological material

Policy information about studies with human participants or human data. See also policy information about sex, gender (identity/presentation), and sexual orientation and race, ethnicity and racism.

Reporting on sex and gender	N/A
Reporting on race, ethnicity, or other socially relevant groupings	N/A
Population characteristics	N/A
Recruitment	N/A
Ethics oversight	N/A

Note that full information on the approval of the study protocol must also be provided in the manuscript.

Field-specific reporting

Please select the one below that is the best fit for your research. If you are not sure, read the appropriate sections before making your selection.

☒ Life sciences ☐ Behavioural & social sciences ☐ Ecological, evolutionary & environmental sciences

For a reference copy of the document with all sections, see nature.com/documents/nr-reporting-summary-flat.pdf

Life sciences study design

All studies must disclose on these points even when the disclosure is negative.

Sample size	Sample sizes were determined according to convention for in vivo experiments (i.e. minimum of two separate biological experiments per condition).
Data exclusions	Data were not excluded from analyses.
Replication	Replication was successful and experiments were performed at least in duplicate.
Randomization	Randomization was not used or relevant to this genomic study. Replicate samples were treated completely separately and used to ascertain reproducibility.
Blinding	Blinding was not relevant to this study, as the data analyses performed here are all genomic analyses.

Reporting for specific materials, systems and methods

We require information from authors about some types of materials, experimental systems and methods used in many studies. Here, indicate whether each material, system or method listed is relevant to your study. If you are not sure if a list item applies to your research, read the appropriate section before selecting a response.

Materials & experimental systems

n/a	Involved in the study	
☐	☒	Antibodies
☐	☒	Eukaryotic cell lines
☒	☐	Palaeontology and archaeology
☐	☒	Animals and other organisms
☒	☐	Clinical data
☒	☐	Dual use research of concern
☒	☐	Plants

Methods

n/a	Involved in the study	
☒	☐	ChIP-seq
☒	☐	Flow cytometry
☒	☐	MRI-based neuroimaging

Antibodies

Antibodies used	SOX2 Cell Signaling Technology 23064S<br>Vinculin Sigma-Aldrich V9264

Validation	Antibodies were validated by manufacturer.

Eukaryotic cell lines

Policy information about cell lines and Sex and Gender in Research

Cell line source(s)	E14 mESCs were gifted from Elphege Nora Laboratory at University of California, San Francisco (UCSF).<br>SOX2-FKBP mESCs were provided by the laboratory of Elzo de Wit (Netherlands Cancer Institute).<br>CTCF-AID mESCs (EN52.9.1) were generated by the laboratory of Elphège Nora (University of California, San Francisco).<br>NIPBL-FKBP mESCs (EA.18.1) were generated by the laboratory of Elphège Nora (University of California, San Francisco).<br>Normal human male iPSC line GM25256 (WTC, hPSCreg: UCSFi001-A) were generated at Gladstone Institutes, distributed by Coriell Institute for Medical Research.<br>mESC lines derived from H1c/H1d/H1e triple knockout (TKO) and wild-type (WT) littermates were generated by the laboratory of Yuhong Fan (Georgia Institute of Technology).
Authentication	SOX2-FKBP mESCs, CTCF-AID mESCs, NIPBL-FKBP mESCs, human iPSC line GM25256, and mESC lines derived from H1c/H1d/H1e triple knockout (TKO) and wild-type (WT) littermates were obtained directly from laboratories that previously published and characterized these lines. E14 mESCs were not authenticated directly, although genotyping data from PacBio sequencing of cells from these line appear concordant.
Mycoplasma contamination	All cell lines used in this study were regularly tested for mycoplasma contamination (Lonza LT07-318) and confirmed to be negative for mycoplasma.
Commonly misidentified lines<br>(See ICLAC register)	No commonly misidentified cell lines were used in this study.

Animals and other research organisms

Policy information about studies involving animals; ARRIVE guidelines recommended for reporting animal research, and Sex and Gender in Research

Laboratory animals	6-week old, C57BL/6J mice (Jackson Laboratory #000664) were used as wild-type hepatocyte samples in our dataset. Foxa2ΔHx-TagRFP-expressing mice were provided by the laboratory of Kenneth Zaret (University of Pennsylvania).
Wild animals	N/A
Reporting on sex	Samples in this study included heterozygous FOXA2-ΔHx mice (n = 3; 1 male and 2 females) and a littermate wild-type control male mouse.
Field-collected samples	N/A
Ethics oversight	All animal experiments were performed under the supervision and approval of the Institutional Animal Care and Uses Committee (IACUC) and the University of California, San Francisco (Protocol # AN179718-03F).

Note that full information on the approval of the study protocol must also be provided in the manuscript.

Plants

Seed stocks	N/A
Novel plant genotypes	N/A
Authentication	N/A
